# Interactions and Cytotoxicity of Human Neurodegeneration- Associated Proteins Tau and α-Synuclein in the Simple Model *Dictyostelium discoideum*

**DOI:** 10.3389/fcell.2021.741662

**Published:** 2021-09-06

**Authors:** Katelyn Mroczek, Sanjanie Fernando, Paul R. Fisher, Sarah J. Annesley

**Affiliations:** Department of Physiology, Anatomy and Microbiology, La Trobe University, Bundoora, VIC, Australia

**Keywords:** tau, α-synuclein, *Dictyostelium*, tauopathies, Alzheimer’s disease

## Abstract

The abnormal accumulation of the tau protein into aggregates is a hallmark in neurodegenerative diseases collectively known as tauopathies. In normal conditions, tau binds off and on microtubules aiding in their assembly and stability dependent on the phosphorylation state of the protein. In disease-affected neurons, hyperphosphorylation leads to the accumulation of the tau protein into aggregates, mainly neurofibrillary tangles (NFT) which have been seen to colocalise with other protein aggregates in neurodegeneration. One such protein is α-synuclein, the main constituent of Lewy bodies (LB), a hallmark of Parkinson’s disease (PD). In many neurodegenerative diseases, including PD, the colocalisation of tau and α-synuclein has been observed, suggesting possible interactions between the two proteins. To explore the cytotoxicity and interactions between these two proteins, we expressed full length human tau and α-synuclein in *Dictyostelium discoideum* alone, and in combination. We show that tau is phosphorylated in *D. discoideum* and colocalises closely (within 40 nm) with tubulin throughout the cytoplasm of the cell as well as with α-synuclein at the cortex. Expressing wild type α-synuclein alone caused inhibited growth on bacterial lawns, phagocytosis and intracellular *Legionella* proliferation rates, but activated mitochondrial respiration and non-mitochondrial oxygen consumption. The expression of tau alone impaired multicellular morphogenesis, axenic growth and phototaxis, while enhancing intracellular *Legionella* proliferation. Direct respirometric assays showed that tau impairs mitochondrial ATP synthesis and increased the “proton leak,” while having no impact on respiratory complex I or II function. In most cases depending on the phenotype, the coexpression of tau and α-synuclein exacerbated (phototaxis, fruiting body morphology), or reversed (phagocytosis, growth on plates, mitochondrial respiratory function, *Legionella* proliferation) the defects caused by either tau or α-synuclein expressed individually. Proteomics data revealed distinct patterns of dysregulation in strains ectopically expressing tau or α-synuclein or both, but down regulation of expression of cytoskeletal proteins was apparent in all three groups and most evident in the strain expressing both proteins. These results indicate that tau and α-synuclein exhibit different but overlapping patterns of intracellular localisation, that they individually exert distinct but overlapping patterns of cytotoxic effects and that they interact, probably physically in the cell cortex as well as directly or indirectly in affecting some phenotypes. The results show the efficacy of using *D. discoideum* as a model to study the interaction of proteins involved in neurodegeneration.

## Introduction

Neurodegenerative diseases are the leading cause of disability worldwide and as the population ages they are expected to increase in their prevalence. The WHO estimate that collectively neurological disorders will eclipse cancer as the second leading cause of death worldwide by 2040 (Gammon, 2014). The most common neurodegenerative disease is Alzheimer’s disease (AD) in which the hallmarks like other neurodegenerative diseases are the accumulation of aggregated proteins. In AD, first described by Alois Alzheimer in 1906, the most abundant aggregated proteins are tau and amyloid beta (Stelzmann et al., 1995). The accumulation of the tau protein is present as the dominant aggregate in a group of neurodegenerative diseases known as tauopathies with AD being the most common tauopathy. However, tau aggregation is also implicated in other diseases including Pick’s disease, Cortico-basal degeneration, Progressive supranuclear palsy and Fronto-temporal dementia with Parkinsonism linked to chromosome 17 (Spillantini and Goedert, 1998; Lee et al., 2001; Goedert and Spillantini, 2011).

Tau is a major mammalian microtubule associated protein (MAP), and in humans it is predominantly found in the neurons (Drubin and Kirschner, 1986). The protein binds to tubulin promoting the assembly and stability of microtubules, supporting axonal transport and structure of neurons (Cleveland et al., 1977; Kosik, 1993). Tau is encoded by the MAPT gene, is 100 kb in size containing 16 exons. Alternative splicing of the RNA transcript at exons 2, 3, and 10 produces six isoforms (Andreadis et al., 1992), which range from 352 to 441 amino acids in length containing either 3 or 4 microtubule binding repeat sequences (Goedert et al., 1989a, b; Himmler et al., 1989). The tau protein contains an acidic N terminal region known as the projection domain, thought to maintain space between microtubules as well as interacting with other cytoskeletal proteins (Avila et al., 2004; Mandelkow et al., 2007). The proline rich middle of the protein binds other proteins and the C terminal contains the microtubule binding domain (MTBD), with either 3 or 4 repeats dependent on the presence or absence of exon 10 (Mandelkow et al., 2007). Four-repeat (4R) tau binds with a higher affinity to microtubules than 3R tau. The longest tau isoform (2N4R) contains both exons 2 and 3 in the N terminus and 4 repeats in the MTBD.

In normal conditions tau binds on and off microtubules depending on the phosphorylation state of the protein. On the 2N4R isoform there have been over 80 phosphorylation sites detected with the majority of these implicated in pathological conditions (Buée et al., 2000; Hanger et al., 2007). Dephosphorylation of tau induces the affinity to bind to MT while phosphorylation leads to a disassociation with MT (Cleveland et al., 1977; Lindwall and Cole, 1984). The further hyperphosphorylation of tau stimulates the accumulation of the protein into aggregates. There are many tau aggregate conformations but the most commonly observed is neurofibrillary tangles (NFT) composed of paired helical filaments (Grundke-Iqbal et al., 1986). Tangles of tau have been pathologically implicated in Alzheimer’s disease, Parkinson’s disease (PD), Down’s syndrome, Parkinsonism–dementia complex of Guam, and progressive supranuclear palsy (Joachim et al., 1987).

The toxic aggregation of tau in diseases like AD, PD, and other tauopathies seems to coincide with mitochondrial impairment associated with transport, dynamics and bioenergetics of the mitochondria (reviewed by Gibson et al., 2010; Eckert et al., 2011; Cabezas-Opazo et al., 2015; Pérez et al., 2018a). Mitochondrial abnormalities include impaired oxidative phosphorylation in a mouse model of AD (Rhein et al., 2009) and impaired mitochondrial dynamics by truncated tau in immortalised cortical neurons and primary cortical neurons from tau knockout mice (Pérez et al., 2018b). The overexpression of tau in neuronal cultures decreased ATP production, the ratio of ATP/ADP and inhibited Complex I activity (Li et al., 2016) and it has been suggested that tau impairs mitochondrial function while simultaneously inhibiting the degradation of damaged mitochondria leading to a cycle of mitochondrial dysfunction (Cummins et al., 2019).

Aggregation and mitochondrial dysfunction are hallmarks of many neurodegenerative diseases. Within the large group of tauopathies the accumulation of proteins other than tau is evident. One such protein is α-synuclein. α-synuclein is encoded by the *SNCA* gene, is 140 amino acids in length and like tau is richly expressed in neurons. Aggregation of α-synuclein and its presence as the dominant protein in Lewy bodies (LB) is a hallmark of a group of neurodegenerative diseases classified as the synucleinopathies. PD is the most common synucleinopathy but other diseases include Lewy Body Dementia, multiple system atrophy, and Parkinsonism with dementia (Galpern and Lang, 2006; Savica et al., 2013). α-Synuclein is the most abundant aggregated protein in these disorders although other proteins like tau have been seen to colocalise within these aggregates as well suggesting an overlap of the tau- and synuclein-opathies.

There have been many neurodegenerative diseases in which tau and α-synuclein have been found to coexist, and several lines of evidence suggest an interaction between the two proteins. Colocalisation of tau and α-synuclein has been seen in both NFT and LB (Arima et al., 2000; Ishizawa et al., 2003). α-synuclein containing LB were seen to be present in AD brains over 50% of the time (Hamilton, 2000), in Down’s syndrome brains with AD 50% of the time (Lippa et al., 1999) and found with PHF of tau in the same neurons (Iseki et al., 1999). The C-terminus of α-synuclein has been seen to bind to the MT Binding Domain of tau promoting phosphorylation by protein-kinase A (Jensen et al., 1999). *In vitro* experiments using human cell lines have shown that tau and α-synuclein can promote the aggregation of each other and increase cytotoxicity (Badiola et al., 2011; Castillo-Carranza et al., 2018). The application of models to study the interaction of the proteins has proved useful and support an interaction. A transgenic mouse model of PD overexpressing α-synuclein increased tau phosphorylation and hyperphosphorylation and aggregates similar to LB were formed containing both tau and α-synuclein (Haggerty et al., 2011). In *Drosophila*, neurotoxic symptoms were enhanced with the coexpression of both proteins compared to when tau or α-synuclein were expressed individually (Roy and Jackson, 2014). In yeast models, the coexpression enhanced aggregation of the proteins, increased phosphorylation of tau and produced toxic effects (Zabrocki et al., 2005; Ciaccioli et al., 2013).

There are many models of tauopathies and using simple models to study complex disease mechanisms has its advantages. *Dictyostelium discoideum* is a novel eukaryotic model organism recognised by the National Institute of Health (NIH) in the United States for its importance in biomedical research. It has been used as a biomedical model for studying human diseases including neurodegeneration, lysosomal trafficking disorders and mitochondrial disease (Annesley and Fisher, 2009a; Francione et al., 2011; Maniak, 2011; Martín-González et al., 2021). The complete nuclear (Eichinger et al., 2005) and also mitochondrial (Ogawa et al., 2000) genomes have been sequenced, allowing for orthologues of human genes to be studied. Of interest is the distinctive life cycle of *D. discoideum* in which there are both unicellular and multicellular stages with numerous cell types. This allows diverse, reproducible phenotypic traits to serve as “readouts” of disease gene-related disturbances in cellular processes and the signalling pathways that control them (Annesley and Fisher, 2009a). Additionally, *D. discoideum* is greatly accessible for use in biomedical research, genetically manipulable and easily grown clonally. Mitochondrial disease has been created and well characterised in *D. discoideum.* Through the knockdown of nuclear encoded mitochondrial proteins or disrupting mitochondrial genes, a clear set of phenotypes was exhibited in each case (reviewed by Francione and Fisher, 2011). Among these phenotypes were: decreased growth on bacterial lawns and in axenic medium, aberrant fruiting body with shorter and thicker stalks, defective slug phototaxis and increased susceptibility to *Legionella* proliferation. These mitochondrially diseased *D. discoideum* phenotypes were attributed to the chronic activation of the energy sensing protein AMP-activated protein kinase (AMPK) by Bokko et al. (2007) and Francione et al. (2009) that showed when AMPK was knocked down by antisense inhibition, the defective phenotypes returned to wildtype levels, and chronic activation of AMPK mimicked the disease phenotypes.

Previously we showed that human α-synuclein can be expressed in *D. discoideum* and has effects on plaque expansion rates, phagocytosis, and PD associated mutations impaired phototaxis. These impaired phenotypes were rescued or partially rescued with the coexpression of an AMPK antisense construct. Suggesting that mitochondrial dysfunction may be attributing to at least some of the defective phenotypes. However, when mitochondrial function was directly measured, α-synuclein displayed an increased mitochondrial respiration rather than a dysfunction (Fernando et al., 2020). This was in line with other PD models in which mitochondrial respiration was seen to be increased including α-synuclein fibrils in neuroblastoma cells (Ugalde et al., 2020), lymphoblast cell lines made from idiopathic PD patients (iPD) (Annesley et al., 2016) and fibroblasts from iPD patients (Haylett et al., 2016). Here we expressed the longest human tau isoform to create a *D. discoideum* model of tauopathies. *D. discoideum* has already been used as a model to study elements involved in AD. Orthologues of the presenilin proteins and other γ secretase subunits that cause the cleavage of amyloid precursor protein (APP) to Aβ have been identified in *D. discoideum* and have similar functionality to the mammalian complex (McMains et al., 2010; Ludtmann et al., 2014). Mutations in the presenilin genes have been known to cause early-onset familial AD (De Strooper and Annaert, 2010). In *D. discoideum*, the knockout of the presenilin genes resulted in a developmental block in aggregation, which was restored with the expression of human presenilin 1 (Ludtmann et al., 2014). Also importantly, *D. discoideum* was seen to process ectopically expressed human APP to the amyloid-β peptides Aβ_40_ and Aβ_42_, while in strains that were deficient in γ secretase this APP processing was blocked (McMains et al., 2010). As is the case with APP, there are no homologues of tau in *D. discoideum*, but many of the proteins that interact with tau have been evolutionarily conserved such as the kinases AMPK and glycogen synthase kinase 3 (GSK3) as well as cytoskeletal proteins tubulin and filamin. This allows the study of the cytotoxic effects of tau directly without the complication of the endogenously expressed protein or homologue.

As there is evidence of an interaction of tau and α-synuclein in neurodegeneration, we also coexpressed human tau and human α-synuclein together to compare to the phenotypes displayed by expressing tau and α-synuclein alone. Tau caused phenotypes similar to those of mitochondrially diseased strains and direct measurements of mitochondrial activity showed a decrease in ATP synthesis. We show here that *D. discoideum* is a viable model to study tauopathies as tau is cytotoxic, can be phosphorylated and is readily coexpressed with other neurodegenerative proteins. The coexpression of human tau and human α-synuclein in this work revealed physical and functional interactions between the two proteins that enhance or reverse their individual cytotoxic effects.

## Materials and Methods

### *Dictyostelium* Strains and Culture Conditions

Wildtype (AX2) and transformed *D. discoideum* strains were grown both axenically and on SM agar plates. Axenically, strains were shaken at 150 rpm at 21°C in HL5 medium containing glucose (Formedium, Hunstanton, Norfolk, United Kingdom), with the addition of 20 μg mL^–1^ geneticin (G418—Thermo Fisher Scientific, Waltham, MA, United States) for transformed strains. They were also grown on SM agar plates (Formedium, Hunstanton, Norfolk, United Kingdom), with *Enterobacter aerogenes* as a food source and supplemented with G418 for transformants. The transformants expressed either human tau (containing plasmid construct pPROF665), human α-synuclein (containing plasmid pPROF629) or were cotransformants expressing both tau and α-synuclein.

### Plasmid Construction

The human tau gene was PCR amplified using a commercially purchased plasmid template tau/pET29b (Addgene plasmid # 16316) containing a cloned copy of the full-length human tau gene (2N4R). Primers Tau2F (CG**A TCG AT**A TGG CTG AGC CCC GC) and Tau2R (CG**C TCG AG**T CAC AAA CCC TGC TTG GC) synthesised by Gene Works Ltd., incorporated *Xho*I (Promega, Madison, WI, United States) and *Cla*I (Promega, Madison, WI, United States) restriction sites and was initially cloned into the *Escherichia coli* vector pCR^®^2.1-TOPO (Thermo Fisher, Waltham, MA, United States, A32728) to produce pPROF664. Subsequently, the Tau insert was subcloned into a *D. discoideum* expression vector by replacing the existing tetracycline cassette in pPROF267. The resultant clone was called pPROF665. The construction of the α-synuclein construct (pPROF629) has been described previously (Fernando et al., 2020).

### Transformation

The transformation of AX2 cells was performed using the calcium phosphate DNA coprecipitation method (Nellen et al., 1984) with 20 μg of construct DNA. Transformants were selected for on SM agar plates containing 20 μg mL^–1^ G418 and a lawn of *Micrococcus luteus* (Wilczynska and Fisher, 1994), the purified transformants were maintained by subculturing in HL5 medium and onto lawns of *E. aerogenes.*

### Quantitative PCR

To quantify the construct copy number in the transformants, quantitative PCR was performed using iQ SYBR Green supermix and an iCycler IQ Multicolor Real-Time PCR Detection system according to manufacturer’s instructions (Bio-Rad, Hercules, CA, United States). The primers used for amplification of a portion of each gene were RTTauF (AAGAGCACTCCAACAGCGGAAGAT) and RTTauR (GTGTCTCCAATGCCTGCTTCTTCA) for tau, and for the α-synuclein gene RTsynF (GCGCTCTAGAATCGATATGGATGTATTCATGAAAGGACT TTCAAAGGCC) and RTsynR (GCGCTCTAGACTCGAGTT AAGGATCCACAGGCATATCTTCCAGAATTCC). The filamin gene was amplified and used as loading control, as it is a single copy gene, and amplified using the primers Fil443For (CCACAGAGATATTGGAGTTGCGTACC) and Fil552Rev (CAACTCAACCAATGTGCCTGCCAA). Two calibration curves were prepared, one to estimate loading using genomic DNA of the wildtype AX2 and the filamin primers to estimate the total quantity of genomic DNA, and the second using purified plasmids to determine the quantity of inserted plasmid constructs. Copy numbers of the inserted constructs for each strain were calculated based on the quantity of the construct, the quantity of gDNA and the sizes (in base pairs) of the amplified fragment and the *Dictyostelium* genome. The copy numbers were then normalised against the copy number of the control AX2 parental strain.

### Western Blotting

An aliquot of 1 × 10^7^ cells of either tau or cotransformant strains as well as the WT were pelleted, supernatant removed and the pellet resuspended in 75 μL of Laemmli 2× sample buffer (4% SDS, 20% glycerol, 0.004% bromophenol blue and 0.125 M Tris-Cl, pH 6.8). Samples were boiled for 10 min and 15 μL loaded to a 10–15% SDS-PAGE gel and transferred to a PVDF membrane (Bio-Rad, Hercules, CA, United States). To detect tau, membranes were incubated with mouse monoclonal anti-tau antibody [Tau-5] (Abcam, Cambridge, United Kingdom, ab80579) (1:2,000), to detect α-synuclein membranes were incubated with mouse monoclonal anti-alpha-synuclein antibody [syn211] (Abcam, Cambridge, United Kingdom, ab80627) (1:2,000) and to detect phosphorylated tau membranes were incubated with rabbit monoclonal recombinant anti-tau (phosphor S404) antibody [EPR2605] (Abcam, Cambridge, United Kingdom, ab92676) (1:500). This was followed by incubation with (goat) anti-mouse or anti-rabbit Cross-Adsorbed Secondary antibody Alexa-Fluor^TM^ 647 (Thermo Fisher Scientific, Waltham, MA, United States, A32728) (1:1,000). Proteins were visualised using the STORM image analyser.

### Multicellular Development

*Dictyostelium discoideum* wildtype and transformant cells were grown on SM plates containing a lawn of *E. aerogenes* at 21°C until multicellular fruiting bodies were observed. The fruiting bodies were examined using an Olympus S761TM dissecting microscope and images were captured using a digital Moticam 2300TM camera. Photographs were taken from above the plates to gain an overall view of the population as well as pictures of singular fruiting bodies. This was obtained by slicing out a thin section of agar and placing it on its side so that the entire fruiting body could be seen.

### Growth on Bacterial Lawns

A scraping of wildtype and transformed amoebae were inoculated into the centre of four normal agar [20 g L^–1^ agar (Difco, Detroit, MI, United States); 1 g L^–1^ peptone (Oxoid, Basingstoke, United Kingdom), 1.1 g L^–1^ anhydrous glucose, 1.9972 g L^–1^ KH_2_PO_4_, and 0.356 g L^–1^ Na_2_HPO_4__2*H*_2_O, pH 6.0] plates containing a lawn of *E. coli* B2 as previously described (Bokko et al., 2007). Plates were incubated at 21°C, the diameter of plaque expansion was measured in mm twice daily over a period of approximately 100 h. The plaque expansion rates for each strain was calculated using the statistical computing and graphics software program “R” by linear regression.

### Growth in Axenic Medium

*Dictyostelium discoideum* cells were axenically grown in HL5 until exponential phase was reached, then inoculated into 50 mL of HL5 in 150 mL flasks to a final density of 1 × 10^4^ cells mL^–1^. Cells were incubated at 21°C on an orbital shaker (Ratek, Boronia, VIC, Australia) at 150 rpm over a period of 100 h. Cells were counted twice daily using a haemocytometer (Bright Line, 0.1 mm deep) and the generation times of each strain was calculated in R by log linear regression during exponential growth phase.

### Phagocytosis Assay

The *E. coli* strain expressing the fluorescent protein DsRed (Maselli et al., 2002) was used to measure bacterial uptake of wildtype and transformed cells using the previously described method (Bokko et al., 2007). Cells were grown axenically in HL5, 5 × 10^6^ cells were harvested, washed, and resuspended in 1 mL of phosphate buffer. Cells were starved for 30 min at 21°C with shaking at 150 rpm, then 1 mL of *E. coli* DsRed suspension was added to each sample, and 400 μL of cells were taken in duplicate at time points T = 0 min and T = 30 min. Cells were washed by centrifugation to remove surface bound *E. coli* using 5 mM sodium azide (Sigma-Aldrich, St. Louis, MI, United States) and lysed using 2 mL of 0.25% (v/v) Triton-X-100 in 100 mM Na_2_HPO_4,_ pH 9.2. The hourly rate of bacterial consumption was determined by taking fluorescence measurements at both time points using a Special Module (530 nm excitation and 580 nm emission) in a Modulus Fluorometer (Turner Biosystems, Sunnyvale, CA, United States). The increase in fluorescence over 30 min, the amoebal density and the fluorescence signal per million bacteria (separately determined) were used to calculate the rates of bacterial consumption.

### Pinocytosis Assay

The Fluorescein Isothiocyanate (FITC)-dextran (Sigma-Aldrich, St. Louis, MI, United States, average mol. mass 70 kDa) method was used for measuring liquid uptake as previously described (Klein and Satre, 1986). Vegetative cells were harvested to a density of 1 × 10^7^ cells, resuspended in 1 mL of HL5 and starved for 30 min at 21° shaking. FITC-dextran was added to a concentration of 2 μg mL^–1^ and incubated for 70 min. Cell samples (200 μL) were taken in duplicate at T = 0 min and T = 70 min, pelleted by centrifugation, washed twice in Sorenson buffer and lysed in 2 mL of 0.25% (v/v) Triton-X-100 in 100 mM Na_2_HPO_4,_ pH 9.2. Fluorescence was measured in the Modulus Fluorometer using the Green Module (525 nm excitation and 580–640 nm emission). The hourly rate of uptake of the medium was calculated using a calibration curve relating to fluorescence signal to volume of fluorescence medium, the cell density and the increase in fluorescence over 70 min.

### Legionella Infection Assay

*Legionella pneumophila* infection and proliferation rates in *D. discoideum* was measured as previously described (Francione et al., 2009) and was adapted from Hagele et al. (2000) and Otto et al. (2004). Wildtype and transformant strains were grown to exponential phase axenically in HL5 at 25°C with shaking. Cells were pelleted, washed twice in Sorensen 1× C buffer (17 mM KH_2_PO_4_/Na_2_PO_4_, 50 μM CaCl_2_, pH 6.0) at 600 × *g* for 3 min and resuspended in modified broth (MB) medium (0.7% yeast extract, 1.4% proteose peptone, 0.062% Na_2_HPO_4_.2H_2_O, 0.049% KH_2_PO_4_, pH 6.9) at 5 × 10^5^ cells mL^–1^. Each suspension was aliquoted into 5 wells of a 96 well tissue culture plate to a final density of 1 × 10^5^ cells to be used at a series of time points (0, 24, 48, 72, and 96 h) along with a negative control of only MB, and allowed to adhere for 30 min at 21°C.

The *L. pneumophila* strains used were derivatives of the pathogenic Corby strain (Mampel et al., 2006) and grown on buffered charcoal yeast extract (BCYE) agar containing 5 μg mL^–1^ chloramphenicol at 37°C with 5% CO_2_ atmosphere for 3 days. After this time, the bacteria were harvested and resuspended in distilled water and used to infect the *D. discoideum* strains at a multiplicity of infection (MOI) of 1:1. This was achieved by reading the OD of the bacterial suspension, assuming that an OD_600_ of 1 equates to 10^9^ bacteria mL^–1^. Initial adherence of the *L. pneumophila* to *D. discoideum* cells was performed by centrifugation at 1,370 × *g* for 10 min. At each time point 50 μg mL^–1^ gentamycin sulphate was added to each well for 30 min to kill the extracellular *L. pneumophila.* Then cells were resuspended and pelleted at 13,000 × *g* for 12 min in a microcentrifuge, washed twice and resuspended in Sorenson 1× C buffer. The cells were lysed with 0.02% (w/v) Saponin and vortexed vigorously to release the intracellular *L. pneumophila*. A 10-fold dilution series of the harvested bacteria was prepared up to 1,000-fold for the first two time points and from 10^–1^ to 10^–5^ for time points 48 h and over. This was plated onto BCYE agar plates and incubated at 25–26°C with 5% CO_2_ for 7 days in order to determine the colony-forming units (c.f.u.).

### Phototaxis and Thermotaxis Assays

Qualitative phototaxis and quantitative photo-and-thermotaxis were performed as previously described (Annesley and Fisher, 2009b). Qualitative phototaxis involved scraping the edge of *D. discoideum* plaques growing on *E. aerogenes* lawns and inoculating onto the centre of charcoal agar plates [5% activated charcoal (Sigma-Aldrich, St. Louis, MI, United States), 1.0% agar technical (Thermo Fisher, Waltham, MA, United States)].

For quantitative phototaxis and thermotaxis amoebae were harvested from mass plates and repeatedly washed in cold sterile saline, followed by centrifugation (600 × *g* for 5 min) to remove any bacterial cells. Quantitative phototaxis involved creating dilutions of the cell pellet with cell densities ranging from 6 × 10^4^ to 1.5 × 10^6^. Twenty microlitre of each dilution was plated in duplicate onto charcoal agar plates in a 1 cm^2^ area in the middle of the plate. Both qualitative and quantitative phototaxis plates were incubated at 21°C for 48 h with a single lateral light source to allow slug trails to form. Cell pellets were diluted to 20% (approximately 3 × 10^6^ cells) for quantitative thermotaxis. A 20 μL aliquot was placed in a 1 cm^2^ area onto the centre of water agar plates (1% agar) and incubated in PVC boxes with no light source on a heat bar producing a 0.2°C cm^–1^ temperature gradient at the agar surface. Plates were placed in duplicate at arbitrary temperature points from 1 to 8 with T1 corresponding to 14°C and increasing in 2°C increments to reach 28°C at T8.

All slug trails were transferred to PVC discs and stained with Coomassie blue (Sigma-Aldrich, St. Louis, MI, United States) before being digitised. Qualitative digitising was performed by tracing entire slug trails with a light source at 0°. For quantitative phototaxis and thermotaxis only the beginning and end of the slug trails were digitised and orientation analysed using directional statistics.

### Immunofluorescence

*Dictyostelium discoideum* transformed and wildtype cells were grown axenically to a density of 2 × 10^6^ cells mL^–1^ and then transferred onto sterile coverslips in six-well Costar plates (Nunc^TM^). The cells were allowed to settle for 30 min before the media was replaced with Lo-Flo HL5 (3.85 g L^–1^ glucose, 1.78 g L^–1^ proteose peptone, 0.45 g L^–1^ yeast extract, 0.485 g L^–1^ KH_2_PO_4_, and 1.2 g L^–1^ Na_2_HPO_4_⋅12H_2_O; filter sterilised) for 1 h to equilibrate the cells. The media was aspirated off and the coverslips rinsed in PBS [12 mM Na_2_HPO_4_, 12 mM NaH_2_PO_4_ (pH 6.5)] then cells were fixed with the addition of prechilled (-20°C) methanol/acetone solution (1:1) and left at −20°C for 10 min and again rinsed in PBS.

The coverslips were then incubated with mouse monoclonal anti-tau antibody [Tau-5] (Abcam, Cambridge, United Kingdom, ab80579) or a combination of Tau-5 and rabbit monoclonal anti-alpha + beta synuclein antibody [EP1646Y] (Abcam, Cambridge, United Kingdom, ab51252) diluted (1:100) in blocking buffer [1% (w/v) bovine serum albumin (BSA) and 1% (v/v) cold-water fish skin gelatin in PBS] for 1 h. Coverslips were rinsed in PBS with 0.1% (w/v) BSA twice and incubated with secondary antibody [goat anti-mouse Alexa-Fluor594 (Molecular Probes^TM^ Invitrogen^TM^) or a combination of Alexa-Fluor 594 (goat) and anti-rabbit Alexa-Fluor—488 conjugated IgG antibody (Thermo Fisher Scientific, Waltham, MA, United States, A11034) for cotransformants], diluted (1:500) in PBS with 1% (w/v) BSA and incubated for 45 min in the dark. The coverslips were rinsed in PBS, then DAPI diluted (1:1,000) in PBS was added and incubated for 5 min. The cells were rinsed in PBS then MilliQ H_2_O drained and mounted onto a microscope slide with Ultramount No. 4 (Fronine Laboratory Supplies) and left to dry for 4 h or overnight before visualisation. The cells were detected on an Olympus (Shinjuku City, Tokyo, Japan) BX61T fluorescent microscope and images were digitally captured with the use of an Olympus DP80 camera.

### Duolink^®^

*Dictyostelium discoideum* transformed and wildtype cells were axenically grown to a density of 2 × 10^6^ cells mL^–1^. Coverslips were lined with a Mini PAP pen (Invitrogen^TM^) to create a 1 cm^2^ area then cells were adhered and fixed to the coverslips as described in the previous section. To detect protein interactions using IF techniques, the Duolink^®^ system was used as per manufacturer’s instructions.

Cells were stained in combinations using mouse monoclonal anti-tau antibody Tau-5, rabbit monoclonal anti-tau antibody (E178) (Abcam, Cambridge, United Kingdom, ab32057), rabbit monoclonal anti-alpha + beta synuclein antibody [EP1646Y] and mouse monoclonal anti-alpha-tubulin antibody [12G10] (developed by Frankel & Nelsen, Developmental Studies Hybridoma Bank, created by the NICHD of the NIH and maintained at The University of Iowa, Department of Biology, Iowa City, IA, United States, 52242). The protein-protein interaction was observed (as red dots) using secondary proximity probes, anti-Rabbit MINUS and anti-Mouse PLUS Duolink^TM^ *in situ* detection kit (Sigma-Aldrich Co. LLC) according to the manufacturer’s instructions. An additional Alexa-Fluor 488 conjugated secondary antibody was added in with the proximity probes at times to visualise tubulin.

### Seahorse Respirometry

Mitochondrial respiration of *D. discoideum* wildtype and transformed strains were measured using the Seahorse Extracellular Flux Analyser (Seahorse Biosciences, North Billerica, MA, United States) as described previously (Lay et al., 2016). A combination of drugs were used in sequential order to measure the Oxygen Consumption Rate (OCR) of specific elements of mitochondrial respiration [10 μM DCCD (N,N0-dicyclohexylcarbodimide, an ATP synthase inhibitor (Sigma-Aldrich, St. Louis, MI, United States), 10 μM CCCP (carbonyl cyanide 3-chlorophenol hydrazone, a protonophore (Sigma-Aldrich, St. Louis, MI, United States)], 20 μM rotenone [Complex I inhibitor (Sigma-Aldrich, St. Louis, MI, United States)] and either 10 μM antimycin A [Complex III inhibitor (Sigma-Aldrich, St. Louis, MI, United States)] or 1.5 mM BHAM [benzohydroxamic acid, alternative oxidase (AOX) inhibitor (Sigma-Aldrich, St. Louis, MI, United States)]. Axenically grown *D. discoideum* were harvested, washed, then resuspended in SIH medium (Formedium, Hunstanton, Norfolk, United Kingdom) and supplemented with 20 mM sodium pyruvate and 5 mM sodium malate (pH 7.4). Each strain was plated into eight wells of an assay plate precoated in Matrigel, and the cells were left to settle for 30 min. Measurement cycles consisting of 3 min of mixing, 2 min wait and 3 min measurement time were completed before and after each sequentially added drug, at least three cycles per condition. There were eight replicates of a strain for each condition, except for the last in which either BHAM or Antimycin A were added to four replicate wells. The wildtype AX2 was included as a control in every experiment in four replicate wells, and two each for the final condition. From the measurements taken before and after the addition of the pharmacological agents averages of specific components of mitochondrial respiration were able to be calculated.

### Whole Cell Proteomics

For proteomic analysis each sample (5 × 10^6^ cells in 100 μL of PBS) was prepared for analysis by the La Trobe University Comprehensive Proteomics Platform according to the following protocol: Cell pellets were dissolved in digestion buffer (8 M urea, 50 mM NH_4_HCO_3_, 10 mM dithiothreitol) and incubated at 25°C for 5 h. Iodoacetamide (IAA) was added to a final concentration of 55 mM and incubated for 35 min at 20°C in the dark to alkylate thiol groups. The preparation was then diluted to 1 M urea in 25 mM ammonium bicarbonate (pH 8.5) and sequencing-grade trypsin (Promega) was added to a ratio of 1:50 (wt/wt) to the sample and incubated for 16 h at 37°C. The digests were acidified with 1% (v/v) trifluoroacetic acid (TFA), dried in a SpeedVac centrifuge followed by a desalting step on SDB-XC StageTips (Empore, SDB-XC reversed-phase material, 3M, St. Paul, MN, United States). Briefly: digested proteins were resuspended in 100 μL of 1% (v/v) formic acid and centrifuged at 14,000 rpm for 2 min. The solid-phase extraction was performed according to Rappsilber et al. (2007) with the following modifications: the membrane was conditioned with 50 μL of 80% (v/v) acetonitrile, 0.1% (w/v) trifluoroacetic acid, and then washed with 50 μL of 0.1% trifluoroacetic acid before the tryptic peptides were bound to the membrane. The bound peptides were eluted by 50 μL 80% (v/v) acetonitrile, 0.1% (w/v) trifluoroacetic acid, and dried in a SpeedVac centrifuge.

Peptides reconstituted in 0.1% TFA and 2% acetonitrile (ACN) were loaded using a Thermo Fisher Scientific^TM^ UltiMate^TM^ 3000 RSLCnano system onto a trap column (C18 PepMap 300 μm ID × 2 cm trapping column, Thermo Fisher Scientific) at 15 μL min^–1^ for 6 min. The valve was then switched to allow the precolumn to be in line with the analytical column (Vydac MS C18, 3 μm, 300 Å, and 75 μm ID × 25 cm, Grace Pty. Ltd.). The separation of peptides was performed at 300 nL min^–1^ at 45°C using a linear ACN gradient of buffer A (water with 0.1% formic acid, 2% ACN) and buffer B (water with 0.1% formic acid, 80% ACN), starting at 5% buffer B to 45% over 105 min, then 95% B for 5 min followed by an equilibration step of 15 min (water with 0.1% formic acid, 2% ACN). Data were collected on an Orbitrap Elite (Thermo Fisher Scientific) in Data Dependent Acquisition mode using m/z 300–1,500 as MS scan range, CID MS/MS spectra were collected for the 10 most intense ions at performed at a normalised collision energy of 35% and an isolation width of 2.0 m/z. Dynamic exclusion parameters were set as follows: repeat count 1, duration 90 s, the exclusion list size was set at 500 with early expiration disabled. Other instrument parameters for the Orbitrap were the following: MS scan at 120,000 resolution, maximum injection time 150 ms, AGC target 1 × 10^6^ for a maximum injection time of 75 ms with AGT target of 5000.

The spectra obtained from the instrument were used to search against *Dictyostelium* database (February 2018), together with common contaminants using the Mascot search engine (Matrix Science Ltd., London, United Kingdom). Briefly, carbamidomethylation of cysteines was set as a fixed modification, acetylation of protein N-termini, methionine oxidation was included as variable modifications. Precursor mass tolerance was 10 ppm, product ions were searched at 0.5 Da tolerances, minimum peptide length defined at 6, maximum peptide length 144, and peptide spectral matches (PSM) were validated using Percolator based on q-values at a 1% false discovery rate (FDR).

### Quantification and Statistical Analysis

Proteomics data was analysed using Scaffold (Proteome Software) before exporting data to Excel for further analysis. Proteins detected in fewer than five samples were excluded from analysis. Intensity-Based Absolute Quantitation abundance values were normalised for each set of transformants to the mean total abundance of the parental strain AX2. Data was exported to Excel in which genes/proteins were assigned to one of either two groups on the basis of whether they were significantly up or significantly down compared to the WT using the *p*-values of a two sample *t*-test. Gene enrichment in biological functions and cellular components associated with these resultant gene lists were determined in FunRich software using hypergeometric analysis and Bonferroni method to gain *p*-values. Lists were also entered into the STRING database for a visual representation and false discovery rates of biological processes.

## Results

### Human Tau Can Be Expressed in *D. discoideum* Alone and in Combination With Human α-Synuclein and Is Phosphorylated

**FIGURE 1:**
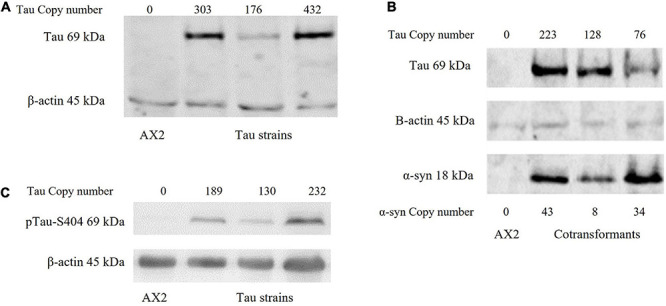
Western blot of strains. **(A)** Human tau can be expressed in *D. discoideum*. A western blot showing the expression of the tau protein in *D. discoideum*. The parental strain AX2 was used as a negative control and β-actin as the loading control. Tau construct copy numbers as estimated by qPCR are indicated at the top of the figure. **(B)** Tau can be expressed in combination with α-synuclein in *D. discoideum*. A western blot showing the expression of both tau and α-synuclein in cotransformant strains. The parental strain AX2 was used as a negative control and β-actin as the loading control. Construct copy numbers are displayed for both tau and α-synuclein. Tau runs at 69 kDa while α-synuclein runs at 18 kDa. **(C)** Tau is phosphorylated in *D. discoideum*. One of the phosphorylation sites implicated in pathological accumulation and aggregation of the tau protein is S404 (Augustinack et al., 2002). An antibody against phosphorylation at this site was used and indicated that tau can be phosphorylated in *D. discoideum*. Construct copy numbers are displayed at the top of each lane. β-actin was used as the loading control and the parental strain AX2 as the negative control.

The longest human tau isoform (2N4R) was expressed in *D. discoideum* singly and in combination with human α-synuclein. The plasmid expression constructs enter the *D. discoideum* genome through rolling circle replication and recombination, this results in strains with a varying number of the construct and therefore different expression levels. Tau phosphorylation plays a major role in the aggregation of the tau protein found in neurodegenerative diseases. In normal circumstances tau binds on and off MT depending on the phosphorylation state of the protein. Phosphorylation causes tau to disassociate from MT while regained affinity to bind to MT occurs with dephosphorylation (Cleveland et al., 1977; Lindwall and Cole, 1984). Further hyperphosphorylation of the protein leads to aggregation and the pathological conformations of tau seen in the tauopathies (Grundke-Iqbal et al., 1986). *D. discoideum* has homologues of many of the kinases that phosphorylate tau in the human brain and as evident in Figure 1, human tau is phosphorylated at least at residue S404 in *D. discoideum.*

### Tau Is Localised Throughout the Cytoplasm in *D. discoideum* Whereas α-Synuclein Is Localised to the Cortex

**FIGURE 2:**
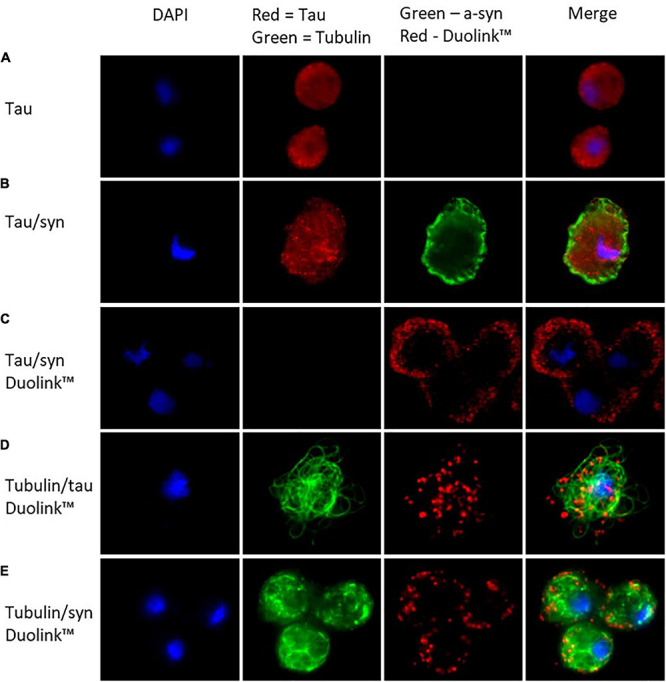
In *D. discoideum* tau localises throughout the cytoplasm of the cell while α-synuclein concentrates at the cortex. The colocalisation of the two proteins takes place at the cortex of the cell where α-synuclein is most concentrated. **(A)** Tau was detected in the tau transformant using an anti-tau primary antibody and observed using Alexa-Fluor 594 conjugated secondary antibody. **(B)** α-synuclein was detected using an anti-α-synuclein antibody and visualised using Alexa Fluor 488 conjugated secondary antibody along with tau in the cotransformant. **(C)** To view colocalisation of tau and α-synuclein the cotransformants were stained with mouse anti-tau antibody and rabbit anti-α-synuclein antibody, the protein–protein interaction was observed (as red dots) using secondary proximity probes, anti-rabbit MINUS and anti-mouse PLUS in the Duolink^*textrmTM*^ in situ detection kit (Sigma-Aldrich). To visualise colocalisation of tau or α-synuclein with tubulin Duolink^*textrmTM*^ was again used, this time using a mouse-anti-tubulin primary antibody and rabbit-anti-tau or anti-α-synuclein antibodies. An additional Alexa-Fluor 488 conjugated secondary antibody was added in with the proximity probes in order to visualise tubulin. **(D)** Tau colocalised with tubulin throughout the cytoplasm of the cell. **(E)** The colocalisation of tubulin and α-synuclein occurred at the cortex of the cell where α-synuclein is most abundant.

To visualise the localisation of tau in *D. discoideum* immunofluorescence microscopy was performed. Tau was detected using an anti-tau antibody coupled with Alexa-Fluor 594-conjugated secondary antibody and was seen throughout the cytoplasm of the cell. α-synuclein had previously been seen to localise to the cortex of the cell in *D. discoideum* (Fernando et al., 2020). To detect α-synuclein and tau in the cotranformants anti-tau and anti-α-synuclein antibodies were coupled with Alexa-Fluor 594 and 488-conjugated secondary antibodies respectively. In the cotransformants tau was again located throughout the cytoplasm and α-synuclein primarily at the cortex. Tau and α-synuclein have been found to colocalise in many neurodegenerative diseases where they may enhance the pathological process of the other protein. To investigate whether tau and α-synuclein colocalise within *D. discoideum*, proximity ligation assays (PLA) were performed using the Duolink^TM^ *in situ* protein-protein interaction detection assay. Tau and α-synuclein were found to colocalise at the cortex of the cell where α-synuclein is primarily concentrated (Figures 2A–C).

### Human Tau and α-Synuclein Can Interact With *D. discoideum* Tubulin

In humans, tau binds to microtubules aiding in their assembly and stability and supporting axonal transport. To determine whether tau interacts with tubulin in *D. discoideum* the Duolink^TM^ detection assay was again used. Tau was seen to localise with tubulin throughout the cytoplasm of the cell (Figure 2D). Tubulin and α-synuclein were also seen to colocalise, however, this interaction was seen at the cortex of the cell where α-synuclein is primarily located (Figure 2E).

### Tau Negatively Affects Fruiting Body Morphology

**FIGURE 3:**
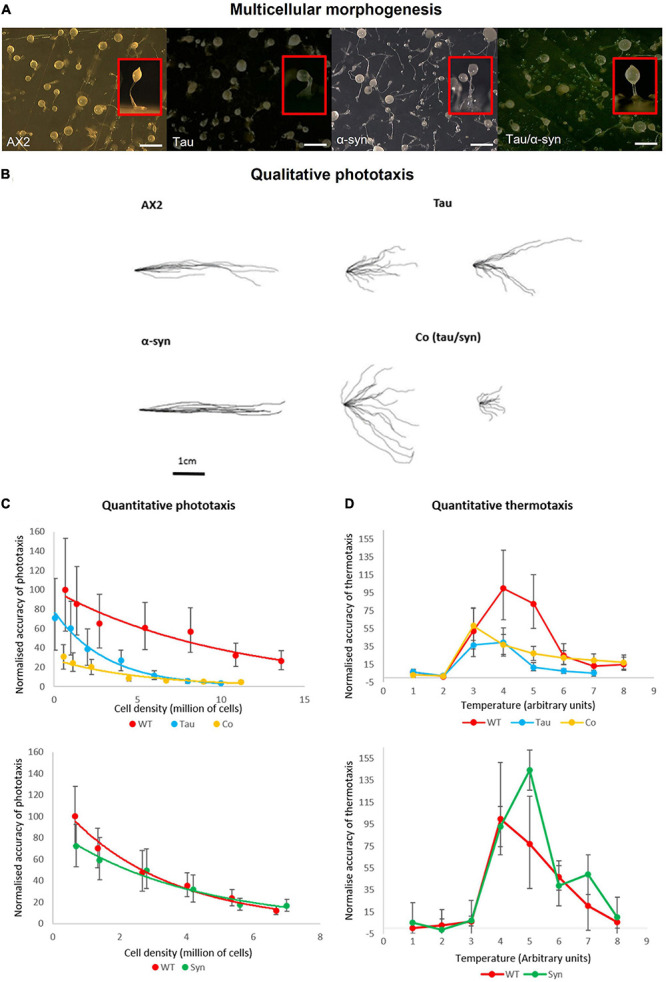
Multicellular morphogenesis, phototaxis and thermotaxis. **(A)** Fruiting body morphology by AX2, strains expressing tau and α-synuclein singularly and coexpression of the two proteins. Tau strains produce an abnormal stalk with enlarged basal disk when compared to the parental strain. The α-synuclein strains resemble that of the parental strain AX2 (Fernando et al., 2020). The strains expressing both tau and α-synuclein have a thicker stalk and larger sorus indicating that these strains produce larger aggregates than AX2. Top view of plates with insets showing the side view of a single representative fruiting body of each strain. Scale bar indicates 1 mm. **(B)** Qualitative phototaxis. Slug trails of WT AX2, tau, α-synuclein, and cotransformants were traced and digitised. Strains expressing α-synuclein showed no defect in phototaxis when compared to WT. Tau strains exhibited a reduced accuracy of phototaxis which was enhanced by the coexpression with α-synuclein. **(C)** Quantitative Phototaxis. WT AX2, tau, α-synuclein, and cotransformant strains were statistically analysed to measure the accuracy of phototaxis (κ), normalised, and were plotted againt cell density. Tau expressing strains displayed an impaired accuracy of phototaxis when compared with the WT AX2. This defect was more severe in the strains expressing tau and α-synuclein in combination. Error bars represent standard errors **(D)** Thermotaxis. AX2, tau strain, α-synuclein [HPF885—data taken from previous experiments (Fernando et al., 2020)], and cotransformants were measured for accuracy of thermotaxis (κ), normalised and plotted against temperature. Temperature is expressed in arbitrary units (1–8) corresponding to agar surface temperatures of 14–28°C. α-synuclein caused no thermotaxis defect in comparison to the WT. The tau and cotransformant strains displayed reduced accuracy of thermotaxis, however, there was no significant difference between strains. Error bars represent standard errors.

Loss of a food source induces chemotaxis among unicellular *D. discoideum* leading to aggregation and multicellular development culminating in the development of a fruiting body consisting of a sorus of spores atop a slender stalk. Fruiting body morphology previously seen in α-synuclein expressing strains resembles that of the parental strain AX2 (Fernando et al., 2020). The expression of tau, however, produces an abnormal stalk with enlarged basal disk when compared to the parental strain (Figure 3A). Cells in the basal disk have undergone a programmed cell death and the result suggests that expression of tau increases the number of cells entering this pathway. This phenotype was observed in *D. discoideum* strains characterised as mitochondrially diseased. The strains expressing both tau and α-synuclein have a larger sorus and thicker stalk, suggesting that they form larger aggregates (Figure 3A).

### Tau Causes an Impairment in Phototaxis and Thermotaxis, Which Is Enhanced by the Coexpression With α-Synuclein

Qualitative and quantitative phototaxis experiments were performed with tau strains and cotransformants (Figures 3B,C). In agreement with previous studies WT α-synuclein expressing strains exhibited no phototaxis defect when compared with the wildtype (Fernando et al., 2020). The tau strains displayed a mild defect in accuracy of phototaxis which was more severe in the strains expressing both tau and α-synuclein. The increased severity of the phototaxis defect when both tau and α-synuclein were expressed together hints at an interaction between the two proteins. This is consistent with yeast models of a synergistic relationship between the two proteins, with enhanced defects when both proteins are expressed (Zabrocki et al., 2005; Ciaccioli et al., 2013). In *D. discoideum*, phototaxis and thermotaxis pathways share many downstream genes (Darcy et al., 1994), so that when a phototaxis defect is detected, a thermotaxis defect is usually also present. In support of this, strains expressing α-synuclein displayed no defect in thermotaxis with accuracies similar to AX2 and strains expressing tau displayed reduced accuracies of thermotaxis (Figure 3D). However, cotransformants expressing both tau and α-synuclein showed reduced accuracies of thermotaxis at a similar magnitude to the tau expressing strains suggesting that α-synuclein imparts no additional defect as was seen in phototaxis.

### Coexpression of Tau and α-Synuclein Positively Affects Growth on Plates but Negatively Affects Axenic Growth

**FIGURE 4:**
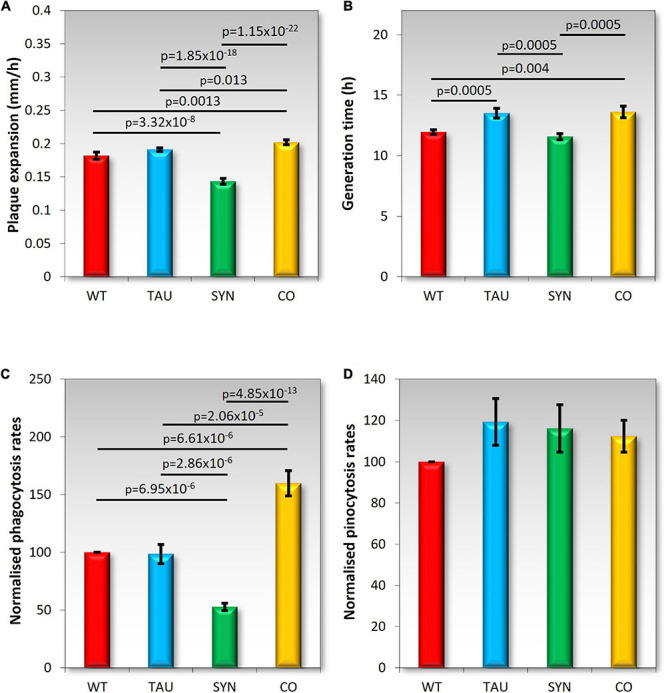
Growth and endocytosis Wild type AX2 (WT) and strains expressing tau (TAU), α-synuclein (SYN), or both tau and α-synuclein (CO) were tested. **(A)** Plaque expansion rates were measured during growth on bacterial lawns of *E. coli* B2. Plaques were measured twice daily over one hundred hours. Experiments were performed in triplicate in four different experiments. As previously seen (Fernando et al., 2020) α-synuclein-expressing strains display slower growth on bacterial lawns when compared to the WT AX2. Tau does not affect growth on plates, but strains expressing both tau and α-synuclein grew slightly but significantly faster. **(B)** Axenic growth rates were measured by determining the generation time of strains (doubling in exponential growth phase). Strains were grown in HL5 liquid medium on a shaker at 21°C for 100 h. Experiments were performed on tau and cotransformant strains over four separate experiments. As previously shown, α-synuclein did not affect axenic growth (Fernando et al., 2020). Strains expressing tau displayed significantly longer generation times during growth in liquid medium. Strains expressing both tau and α-synuclein also showed significantly longer generation times than AX2. Error bars are standard errors of the mean, p-values represent statistically significant values using a two sample *t*-test. **(C)** Phagocytosis rates were measured by feeding *D. discoideum* amoeba *E. coli* DS-Red and taking fluorescent measurements directly after addition of the bacteria, then again after 30 min of incubation at 21°C on a shaker. The uptake rates of bacteria were normalised to the rate of uptake of AX2 (WT). Tau did not affect phagocytosis rates and shows a similar uptake rate to AX2. Previously, α-synuclein expression caused a significant decrease in phagocytosis rates (two-sample *t*-test pictured). The coexpression of tau and α-synuclein significantly increased the rate of phagocytosis (two-sample *t*-test). Cell lines were assayed in at least three separate experiments **(D)** Pinocytosis rates were measured by feeding *D. discoideum* amoeba with HL5 that contained FITC-dextran. Fluorescent measurements were taken directly after the addition of the FITC-dextran and again after an incubation period of 70 min at 21°C on a shaker. Cell lines were assayed in at least three separate experiments. Pinocytosis rates were not significantly affected by the expression of tau, α-synuclein (Fernando et al., 2020), or the combined expression of these two proteins.

*Dictyostelium discoideum* strains expressing tau and α-synuclein alone and in combination were grown on lawns of *E.coli* B2 to measure plaque expansion rates. Previous experiments showed a decrease in plaque expansion rates and no affect on axenic growth by α-synuclein expressing strains (Fernando et al., 2020). Tau strains produced plaque expansion rates similar to AX2, while the cotransformant had slightly faster plaque expansion rates (Figure 4A). This could indicate an interaction between tau and α-synuclein that rescues the α-synuclein defect. Axenic growth was slightly impaired in both tau and cotransformant strains with slightly longer generation times (Figure 4B). These results differ from those seen with mitochondrially diseased strains which displayed decreased growth on both solid and in liquid media.

### Combined Expression of Tau and α-Synuclein Positively Affects Phagocytosis Rates While Macropinocytosis Is Not Affected

*Dictyostelium discoideum* consume nutrients through endocytosis. When feeding on bacterial lawns, bacteria are ingested through phagocytosis, and in liquid media nutrients are taken up through macropinocytosis (pinocytosis). The normalised rates of endocytosis can be seen in Figures 4C,D. Tau did not affect phagocytosis or pinocytosis rates, which resembles the phenotypes of mitochondrially diseased cells (Bokko et al., 2007). Phagocytosis but not pinocytosis was affected in α-synuclein strains, indicating the impaired growth on bacterial lawns was due at least partly to a phagocytosis defect. The cotransformants had an increased phagocytosis rate suggesting that the increased growth rate on bacterial lawns was also due to elevated rates of phagocytosis. Pinocytosis was not affected in these strains and therefore not the cause of the impaired axenic growth of the tau and cotransformant strains.

### *Legionella* Proliferation Is Increased in Tau and Cotransformant Strains

**FIGURE 5:**
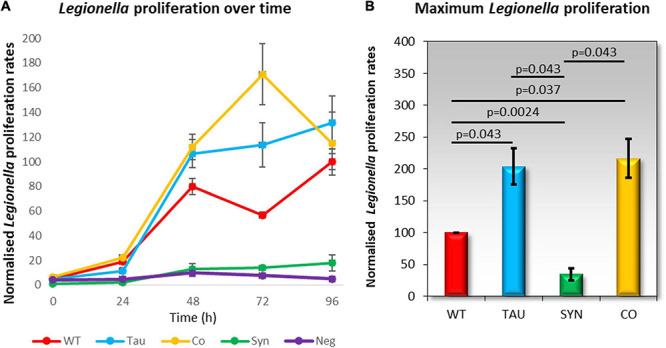
*Legionella* proliferation is increased in tau and cotransformant strains. *Legionella* infection rates were measured by creating a monolayer of *D. discoideum* amoebae in a cell culture plate and infecting these with *L. pneumophila* Corby. The parental strain AX2 (WT), tau expressing strains (TAU), α-synuclein expressing strains (SYN), and cotransformants expressing tau and α-synuclein (CO) were infected with *L. pneumophila* and assayed for viable counts across five days at time periods 0, 24, 48, 72, and 96 h. Extracellular *L. pneumophila* was killed by the addition of Gentamycin sulphate (G418) 30 min before cells were harvested for viable counts. **(A)** The normalised viable counts resulting from the intracellular *L. pneumophila* released by the *D. discoideum* cells were plotted against the corresponding time periods. A negative control was included for comparison and did not contain *D. discoideum* cells. *L. pneumophila* proliferation was increased in tau and co-transformant strains when compared to AX2. The plot values are an average of 3–6 strains per time period and were assayed in at least three individual experiments. The error bars are standard errors of the mean. **(B)** The maximum *L. pneumophila* proliferation rates were normalised to the proliferation in AX2 (WT). *L. pneumophila* proliferation was significantly decreased in α-synuclein expressing strains compared to the WT. There was a significant increase in *L. pneumophila* proliferation in the tau and co-transformant strains in comparison to the parental strain. *p*-Values represent statistical significance using an ANOVA with pairwise comparisons using the Least Squares Difference test. Error bars are the standard errors of the mean.

*Dictyostelium discoideum* is naturally found in soil environments where it consumes bacteria as a food source. *Legionella* also reside in moist soil environments where they can infect and proliferate within amoebae by exploiting phagocytosis. In healthy *D. discoideum* cells, *Legionella* is taken up and proliferates, but in mitochondrially diseased cells, *Legionella* proliferation is enhanced by up to two-fold when compared to wildtype (Francione et al., 2009). To measure *Legionella* infection and intracellular proliferation rates, *D. discoideum* amoebae were plated in a monolayer in a tissue culture plate and infected with *L. pneumophila* Corby. Viable counts of *L. pneumophila* were determined over 5 days at time points 0–96 h. Tau strains and cotransformants exhibited increased *L. pneumophila* proliferation compared to the parental strain AX2 (Figure 5), which corresponds to previous results seen in mitochondrially diseased cells. *D. discoideum* strains expressing α-synuclein showed a decrease in *L. pneumophila* proliferation.

### Phototaxis Defect Is Mediated by AMPK

**FIGURE 6:**
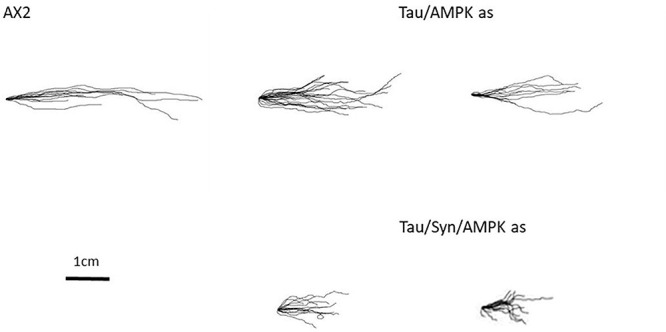
Aberrant phenotypes associated with mitochondrial disease are rescued with antisense inhibition of AMPK. The phototaxis defect is rescued by the knockdown of AMPK. Cotransformants expressing tau and antisense AMPK (Tau/AMPK) rescues the phototaxis defect of tau. The more severe defect that was seen with tau and α-synuclein expression combined was also rescued by antisense inhibition of AMPK (Tau/Syn/AMPK) and strains resemble the WT.

Some of the phenotypes with tau-expressing stains resemble phenotypes attributed to mitochondrial dysfunction in *D. discoideum.* These phenotypes have previously been attributed to the chronic activation of AMPK, an energy-sensing enzyme important in cellular homeostasis. One of the main phenotypes associated with mitochondrial dysfunction and chronic activation of AMPK is a defective slug phototaxis. This was observed in strains in which Cpn60 had been antisense inhibited (Bokko et al., 2007), the mitochondrial protein MidA was knocked out (Carilla-Latorre et al., 2010) and mitochondrial genes were disrupted in a subset of mitochondrial genomes (Francione and Fisher, 2011; Francione et al., 2011). The phototaxis defect in mitochondrially diseased strains is rescued by antisense inhibition of AMPK. Furthermore AMPK has been shown to interact in a photosensory signalling complex with filamin (Bandala-Sanchez et al., 2006) and other proteins implicated in this pathway including RasD (Wilkins et al., 2000), FIP (Knuth et al., 2004), the protein kinases PKB, and ErkB (Bandala-Sanchez et al., 2006). Knockdown of AMPK has also rescued the defective phototaxis seen in strains expressing PD associated mutations of α-synuclein (Fernando et al., 2020). Because of this, we decided to investigate whether the phototaxis defect caused by the expression of tau and the more severe defect of the cotransformant was mediated by increased AMPK activity. To do this, cotransformants were created expressing tau and an AMPK antisense construct. As the expression of tau and α-synuclein combined yielded a more severe phototaxis defect, strains expressing tau, α-synuclein and AMPK knockdown were also produced. Copy numbers of the tau, AMPK antisense and α-synuclein expression constructs were determined by qPCR. AMPK antisense inhibition rescued the phototaxis defect caused by both the expression of tau and the more severe impairment observed in the α-synuclein/tau cotransformants (Figure 6). The slug trails resemble the parental strain in accuracies toward the light source, suggesting that the phototaxis defect in these strains is mediated by chronic AMPK hyperactivity as is known to be the case for mitochondrially diseased strains. It would be of interest in future work to determine if the phenotypes produced by tau that mimic those of mitochondrial disease are also mediated by AMPK. These include the defective fruiting body morphology, axenic growth, thermotaxis, and enhanced *L. pneumophila* proliferation.

### Tau Impairs ATP Synthesis While the Coexpression With α-Synuclein Leads to Normally Functioning Mitochondria

**FIGURE 7:**
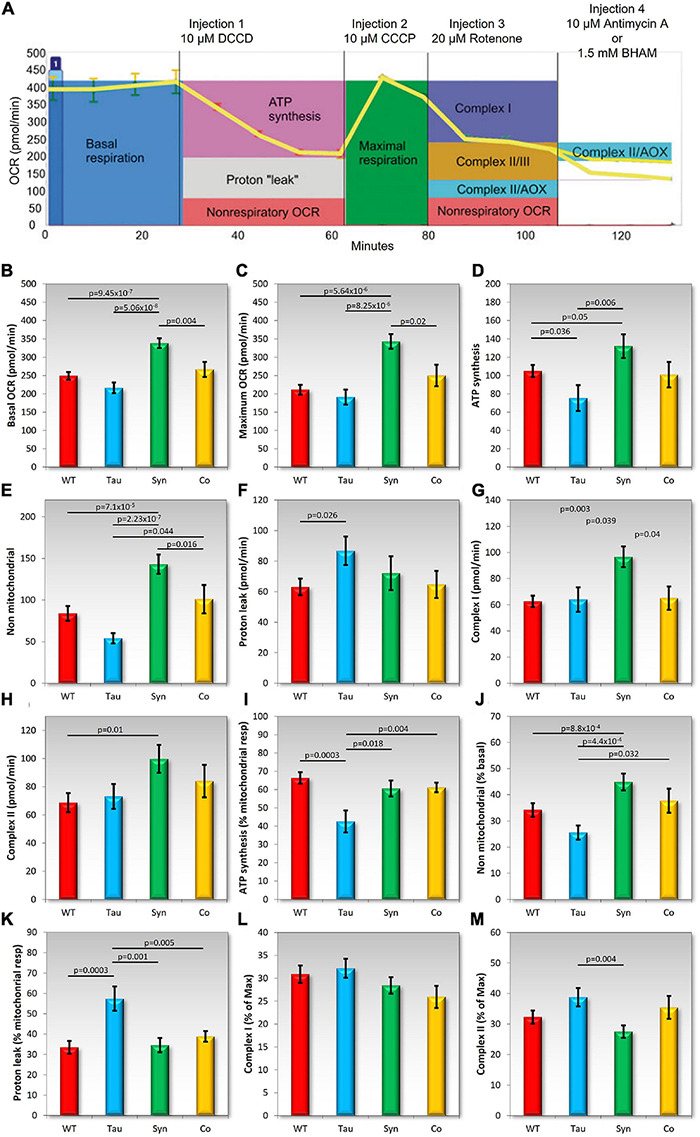
Tau impairs mitochondrial ATP synthesis and coexpression with α-synuclein rescues the defect. In each experiment, cells of the *D. discoideum* parental strain (AX2), and strains expressing tau or α-synuclein alone or in combination were plated in four wells per sample of a Seahorse XFe24 plate. Mitochondrial respiration was measured as the oxygen consumption rate (OCR) using the Seahorse XFe24 Analyzer following the addition of pharmacological agents. The following agents were added sequentially [as seen in panel **(A)** and into all wells: DCCD (dicyclohexylcarbodimide), CCCP (carbonyl cyanide m-chlorophenyl hydrazone), and rotenone]. Then either Antimycin A or BHAM (benzohydroxamic acid) was added to the wells. The coloured boxes in panel **(A)** indicate how each component of the respiratory chain was measured (Lay et al., 2016). Total activity for Complex II was calculated by adding the effects of Antimycin A and BHAM. Panels **(B–H)** represent each component of mitochondrial respiration measured by the OCR. Each strain was assayed as four replicates per experiment across an average of 3–6 experiments. Horizontal bars with *p* values represent statistically significant pairwise comparisons using the *t*-test. All other pairwise differences were not statistically significant. Error bars are a standard error of the mean. The following components were measured: **(B)** Basal OCR, **(C)**, maximum OCR, **(D)** ATP synthesis, **(E)** non-mitochondrial respiration, **(F)** proton leak, **(G)** Complex I activity, and **(H)** Complex II activity. As seen previously, *D. discoideum* expressing α-synuclein showed an increase in mitochondrial respiration and also an increase in oxygen consumption rates as a result of non-mitochondrial processes. In contrast tau expressing strains displayed a reduced OCR dedicated to ATP synthesis **(D)** and an increased proton leak **(F)** and the two appeared to balance each other out as basal respiration was unchanged **(A)** The proportion of ATP synthesis and proton leak to basal respiration was also significantly affected suggesting functional defects in Complex V and proton leak. There were significant differences between tau and α-synuclein expressing strains in the basal and maximum OCRs, ATP synthesis, non-mitochondrial respiration and Complex I activity. In all cases α-synuclein displayed higher OCR measurements than tau strains. The effects on either of these strains seemed to be “balanced” out with the combination of the two proteins being expressed together. The cotransformants showed similar results to the parental strain and did not differ significantly in any component when compared to AX2. **(I–M)** The following were plotted as a proportion of either the Basal, maximum, or mitochondrial respiration dedicated OCR in order to determine the contribution of each component to the relative respiration rates. Shown in these panels are the OCR attributed to ATP synthesis as a % of mitochondrial OCR **(I)**, non-mitochondrial OCR as a % of Basal OCR **(J),** the “proton leak” or the mitochondrial OCR rate not contributed to by ATP synthesis; as a % of mitochondrial respiration **(K),** relative contribution of Complex I activity as a % of Maximum OCR **(L)**, and the relative contribution of Complex II activity as a % of Maximum OCR **(M).** Strains expressing α-synuclein increase in non-mitochondrial OCR as a % of basal. Tau expressing strains displayed a decrease in OCR attributed to ATP synthesis relative to mitochondrial OCR but an increase in mitochondrial OCR attributed to by the “proton leak.” Once again, the cotransformants expressing both tau and α-synuclein did not differ from the parental strain indicating all complexes were functioning normally, making similar relative contributions to respiration as in the parental strain AX2.

The phenotypes caused by ectopic tau expression suggest a possible mitochondrial defect producing at least some AMPK-dependent cytopathological outcomes. Therefore we measured the function of the mitochondria using the Seahorse Extracellular Flux Analyser in combination with a series of inhibiting drugs added in sequential order. This allows the analysis of various components of mitochondrial respiration using the Oxygen Consumption Rate (OCR) as a readout of mitochondrial activity (Figure 7).

Figure 7A shows a typical example of a Seahorse experiment and indicates how each of the components are measured. In agreement with previously conducted experiments, the α-synuclein-expressing strains showed significant increases in mitochondrial respiration and also an increase in the OCR by non-mitochondrial processes. There was no significant difference in the contribution of each component to basal respiration or maximum respiration rates suggesting that all complexes and components are functionally normal but hyperactive, as previously reported. The expression of tau did not affect total mitochondrial respiration but there was a significant decrease in OCR dedicated to ATP synthesis both in absolute terms (pmol min^–1^) and relative to the basal mitochondrial respiration rate. Tau did not affect the maximum uncoupled O_2_ consumption rate, or the contributions to this of Complex I and Complex II. These results suggest a specific defect in Complex V, the mitochondrial ATP synthase. It was accompanied by a significant increase in the O_2_ consumption rate attributable to “proton leak,” both the absolute rate and the proportion it contributed to basal respiration. This could reflect compensatory upregulation of mitochondrial transport processes responsible for provisioning the mitochondria with oxidizable substrates and other molecules. The coexpression of tau and α-synuclein rescued all of these defects and these strains displayed normal mitochondrial function resembling the wild type AX2 strain. This again signifies a functional interaction between the two proteins.

### Conclusions of Proteomics

**FIGURE 8:**
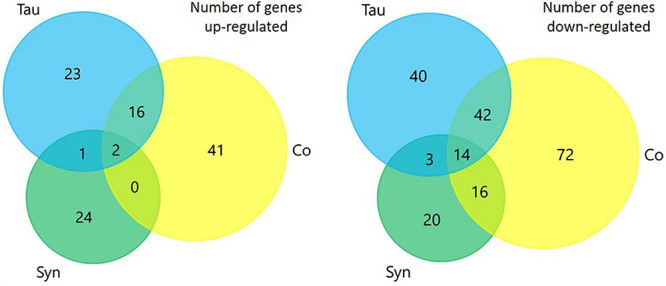
Venn diagrams representing the number of proteins that were differentially expressed in each strain when compared to AX2. There were more proteins down-regulated in each of the sets of strains compared to the number that were up-regulated. The coexpression of tau and α-synuclein lead to the expression of more proteins being up- or down-regulated than either did alone.

To investigate whether any of the phenotypes caused by the expression of tau, α-synuclein or the cotransformant strains were caused by differentially regulated proteins, whole cell proteomics was performed and analysed to compare protein abundances between strains. The number of up- and down-regulated proteins of each strain when compared to AX2 was determined using data exported to Excel from Scaffold and using the *p*-values of two sample *t*-tests. The number of proteins differentially expressed in the tau, α-synuclein, and cotransformant strains can be seen in Figure 8. There were more proteins down-regulated (tau *n* = 99, α-synuclein *n* = 53, cotransformants *n* = 144) in all groups compared to proteins that were up-regulated (tau *n* = 42, α-synuclein *n* = 27, cotransformants *n* = 59), and the cotransformants had more proteins differentially expressed than either the tau or α-synuclein alone.

**FIGURE 9:**
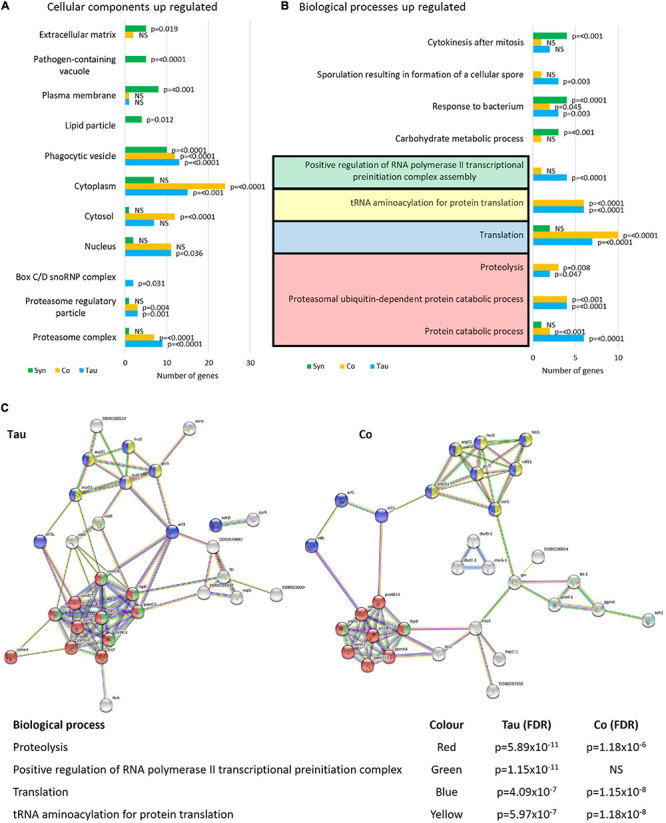
Cellular components and biological processes up-regulated in strains expressing tau and/or α-synuclein. Funrich enrichment analysis of cellular components **(A)** and biological processes **(B)** indicate that tau affects the proteasome and proteolytic processes as well as translation. The expression of α-synuclein resulted in different biological processes and therefore different cellular components being affected. The cotransformants displayed patterns of up-regulated expression similar to those in strains expressing tau alone. The reported *p*-values are based on hypergeometric tests **(A)** and corrected using the Bonferroni method **(B)** calculated in FunRich software. This is based on the number of genes up-regulated within each strain divided by the total number of genes generated for each process/component using the Gene Ontology database for *D. discoideum.* To give a visual representation of protein groups, STRING was used to view the groups of interacting proteins involved in the major up-regulated processes in the tau-expressing strains and cotransformants and calculate False Discovery Rates (FDR) **(C)** which indicate statistical significance of the overrepresentation of these biological processes in the list of interacting up-regulated proteins. There was insufficient data to provide a network of interacting up-regulated proteins in the α-synuclein expressing strains using STRING (Not pictured). Proteins and STRING protein annotations can be found in Supplementary Tables 1, 3, 5.

Enrichment analysis using FunRich software (Figures 9A,B) revealed that of the proteins up-regulated in the tau strains there were significantly more genes affecting protein catabolism (*p* ≤ 0.0001), the proteasome (*p* ≤ 0.0001), and translation (*p* = 0.0001) indicating that protein degradation and synthesis i.e., turnover are up-regulated. α-synuclein did not have any effect on these processes while the cotransformants up-regulated fewer proteins involved in protein catabolism but more in the process of protein synthesis (translation, *p* = 0.0001). Thus, the biological processes up-regulated by α-synuclein were unlike those up-regulated by tau. Tau induces protein catabolism, possibly as part of a spectrum of homeostatic compensatory processes that favour energy production by alternative catabolic processes in the face of defective mitochondrial ATP synthesis. By contrast, α-synuclein expression enhances mitochondrial respiration and perhaps in support of this elevated activity, these strains exhibited higher levels of expression of enzymes involved in carbohydrate metabolism. This may provide the energy to support elevated rates of protein biosynthesis.

In α-synuclein-expressing strains, proteins involved in responding to bacteria were up-regulated as well as proteins associated with the pathogen-containing vacuole (*p* = 0.0001). This accords with the *L. pneumophila* infection and proliferation experiments in which α-synuclein was significantly less susceptible to proliferation when compared with the parental strain AX2. These observations suggest that in α-synuclein-expressing strains, *Legionella*-containing vacuoles may be up-regulated increasing the capacity of the cell to directly handle the pathogens through the endolysosomal *Legionella*-destroying pathway.

In accord with their different subcellular localisations, tau up-regulated proteins in the cytosol and cytoplasm where it was localised (as well as the nucleus), while α-synuclein up-regulated proteins in the plasma membrane, extracellular matrix, and phagocytic vesicle which are all associated with the cell cortex (Figure 9B). STRING was used as a visual representation of the interacting protein groups up-regulated in the tau and cotransformant strains and the False Discovery Rates of each biological process affected (Figure 9C). There were too few up-regulated proteins in the α-synuclein expressing strains for valid comparisons.

**FIGURE 10:**
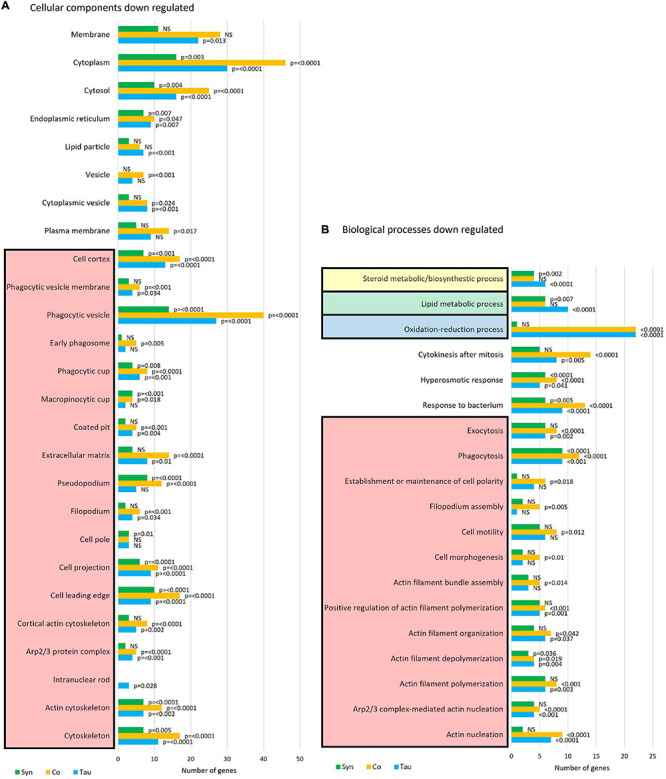
Cellular components and biological processes down-regulated in strains expressing tau and/or α-synuclein. Funrich enrichment analysis of cellular components **(A)** and biological processes **(B)** indicate that the cytoskeleton is significantly down-regulated in all strains. The coexpression of tau and α-synuclein compounded the effect. The reported *p*-values are based on hypergeometric tests using the Bonferroni method of correction calculated in FunRich software. This is based on the number of proteins down-regulated within each strain divided by the total number of proteins generated for each process/component using the Gene Ontology database for *D. discoideum.*

More biological process and cellular components were down- than up-regulated in all three strain groups (Figure 10). Proteins involved with the cytoskeleton were down-regulated in all strains (*p* ≤ 0.0001), with the highest number of proteins down-regulated in strains expressing both tau and α-synuclein. This is another indication of functional interactions between these cytotoxic proteins and is not surprising as the cytoskeleton is involved in many processes that were affected in these strains, including phagocytosis, phototaxis and thermotaxis, differentiation, and development (Noegel and Schleicher, 2000). Of note, the biological processes of cell motility (*p* = 0.012), polarity (*p* = 0.018), morphogenesis (*p* = 0.01), and filopodium assembly (*p* = 0.005) were only significantly down-regulated in the cotransformants. This corresponds with the more severe phototaxis defect in the cotransformants which also displayed a possible motility defect, as the slugs did not travel as far as other strains. It would be of interest to measure single cell motility and chemotaxis in these strains.

Several proteins involved in the response to bacteria are down-regulated in all strain groups, however, this is more significant and there are more proteins affected in the tau-expressing and cotransformant strains (*p* = 0.0001) when compared to those expressing α-synuclein alone (*p* = 0.005). Proteins upregulated in this pathway in the α-synuclein strains were downregulated in the cotransformants. This again relates to the *L. pneumophila* results, wherein the tau and cotransformants both displayed an increased susceptibility to *L. pneumophila* proliferation when compared to the parental strain. Oxidation-reduction processes are significantly down regulated in the tau and cotransformant strains. Most of the proteins involved in these processes are involved in response to oxidative stress and the biosynthetic pathways of amino acid, fatty acid and lipid synthesis.

**FIGURE 11:**
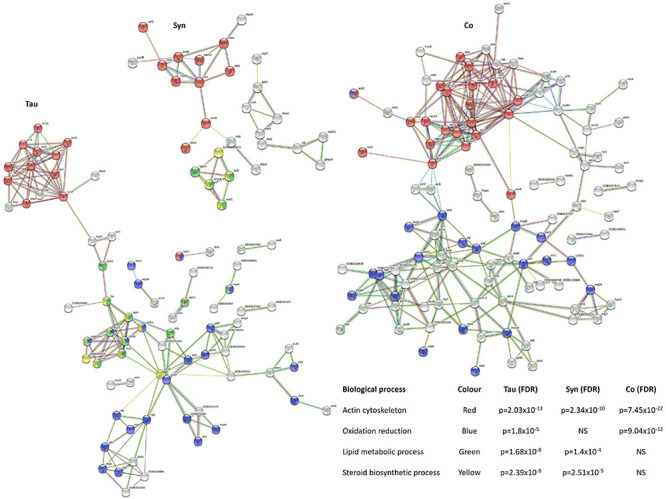
Down-regulated biological processes in strains expressing tau and/or α-synuclein. To give a visual representation of protein groups, STRING was used to view the interacting protein clusters in the main processes down-regulated in the tau, α-synuclein, and cotransformants and calculate False Discovery Rates (FDR) which indicate statistical significance of the overrepresentation of these processes in the list of interacting proteins. Proteins and STRING protein annotations can be found in Supplementary Tables 2, 4, 6.

Lipid metabolism has been found to be dysregulated in association with tau pathology in AD and α-synuclein in PD and synucleinopathies. For reviews see (Bok et al., 2021) and (Alecu and Bennett, 2019). Lewy bodies, the α-synuclein-containing aggregates that are the pathological hallmark of PD, contain high lipid content and lipid membranes (Shahmoradian et al., 2019), while membrane lipids such as those associated with cholesterol have been associated with PHF in AD brains (Gellermann et al., 2006). Here tau and α-synuclein when expressed alone significantly reduced lipid and sterol metabolic pathways, but when coexpressed, the down-regulation was rescued. This resembles the results of mitochondrial respiration where there were effects caused by the single expression of each protein and these were rescued to normal levels with coexpression. Again this signifies a functional interaction between tau and α-synuclein. STRING was used as a visual representation of the protein groups down-regulated in three sets of strains and the False Discovery Rates of each biological process affected (Figure 11).

## Discussion

Tauopathies are a diverse set of neurodegenerative diseases characterised by the accumulation of the tau protein into aggregates. Tau is important in the stabilisation of MT and axonal transport and binds to microtubules based on the phosphorylation state of the protein. Here we expressed the full-length human tau isoform (2N4R) in *D. discoideum* as a model to study tauopathies. As many neurodegenerative diseases including the tauopathies suggest a synergistic relationship between pathological proteins, we also expressed tau in combination with α-synuclein to investigate the interaction between these two proteins.

Phosphorylation of tau leads to disassociation of tau and MT, and further hyperphosphorylation causes the accumulation of tau into aggregates, while dephosphorylation restores tau/MT binding. Tau is regulated by many different kinases and phosphatases (Avila, 2008). Tau has over 80 different phosphorylation sites on the 2N4R isoform and many of these are involved in pathological conditions (Hanger et al., 1998; Buée et al., 2000). Over 20 kinases have been identified which are able to phosphorylate tau. These can be divided into two main groups—the proline directed protein kinases (PDPKs) and non-PDPKs. The PDPKs phosphorylate tau at Serine/Threonine residues and have been linked to the process of neurodegeneration (Sergeant et al., 2008; De Vos et al., 2011). Some common PDPKs are GSK-3β, mitogen activated protein kinase and cyclin dependent kinases which all have homologues in *D. discoideum* (Goldberg et al., 2006*).* These kinases phosphorylate tau at multiple Serine/Threonine sites on the 2N4R isoform (Augustinack et al., 2002). Phosphatases of tau have also been defined and include protein phosphatase 1 (PP1), 2A (PP2A), 2B (PP2B), and 5 (PP5), all of which have homologues in *D. discoideum.* Tau was phosphorylated in *D. discoideum* at S404 which is an important residue in the process of aggregation of the tau protein. Mondragón-Rodríguez et al. (2014) found that the double phosphorylation of Ser396 and Ser404 was an early event in AD and Down’s syndrome. They saw early pathological tau structures (not yet defined NFT) correlating with phosphorylation at Ser396/404, while other phosphorylation sites corresponded with mature NFT. Pseudophosphorylation of tau (achieved by mutating the serine to glutamic acid to mimic phosphorylation) at this epitope has also been found to be an early event in the hyperphosphorylation and aggregation of tau *in vitro* (Abraha et al., 2000; Haase et al., 2004). Phosphorylation specifically at Ser404 has been shown by kinases including GSK-3β and the mitogen activating protein kinase ERK2 (Reynolds et al., 2000). Phosphopeptide mapping and sequencing shows that GSK-3β displays the most pronounced phosphorylation at S404 (Augustinack et al., 2002). Phosphorylation of tau was also seen in other simple models including the yeast model of tauopathy, in which human tau was phosphorylated and dephosphorylated by yeast kinases and phosphatases (De Vos et al., 2011), and site specific phosphorylation resulted in tau aggregates and damage to MT (Vanhelmont et al., 2010). As tau is phosphorylated at S404 in *D. discoideum*, it is likely that it is also phosphorylated at other residues and this could be further investigated. Despite there being no tau orthologue in *D. discoideum*, the presence of homologous kinases that phosphorylate tau and the demonstration that at least one of them does act on tau suggest that the cellular machinery that regulates tau is ancient and could act on it in a similar way in *D. discoideum* as it is in mammals. This is not unlike the case of APP which has no orthologue in *D. discoideum*, but which has been shown to be processed by γ-secretase in *D. discoideum* as in mammalian cells (McMains et al., 2010). As phosphorylation is the first step in the eventual pathological accumulation of tau, the next steps could be to investigate tau aggregation as well as other posttranslational modifications and structural conformations of the protein in *D. discoideum.*

In normal healthy cells, tau binds on and off MT depending on the phosphorylation state of the protein. The longest tau isoform has a high affinity for MT as it has 4 MTBR. Here, tau was localised in the cytoplasm of the cell where it interacted with tubulin, although not necessarily on the MT. As tau was phosphorylated, this could have caused MT disassembly as it does in human neurons. However, as tau is not endogenously expressed in *D. discoideum* it is not necessary for MT stability. In agreement with this, our results provided no evidence of MT disassembly when tau was ectopically expressed in *D. discoideum*, although this was not directly measured.

Ectopic expression of human tau in *Drosophila* revealed that, unlike the endogenous *Drosophila* orthologue, it interacted poorly with *Drosophila* microtubules but was nonetheless cytotoxic (Feuillette et al., 2010). These findings would suggest that tau can exert cytopathological effects that are not related to dysregulated MT assembly/disassembly. What might those processes be? The proteomics results suggested that ectopic tau expression had a major impact on protein turnover with both protein degradation (proteasomal) and protein biosynthesis (transcription and translation) being up-regulated. The phosphorylation state of tau could impact the proteins involved in the proteasome as seen by Ren et al. (2007). Here, tau was expressed in embryonic kidney cells and phosphorylation of tau increased proteasome activity while further hyperphosphorylation decreased activity by the proteasome.

Like tau, α-synuclein has been reported to interact with tubulin heterodimers in the cytosol (Payton et al., 2001) but also with MT, where it altered the cell surface recruitment of the dopamine transporter (Wersinger and Sidhu, 2005). A study by Alim et al. (2002) found that tubulin was a binding partner of α-synuclein and colocalised in LB in a case of PD. However, colocalisation of proteins in the aggregates of Lewy Bodies does not necessarily reflect their normal interactions in the absence of such aggregates. We found that both tubulin and tau colocalise with α-synuclein in *D. discoideum* in the cortex of the cell where α-synuclein is concentrated. It is also here in the cortical regions of the cell that the cytopathological effects of ectopically expressed α-synuclein are exercised—in the inhibition of phagocytosis and *L. pneumophila* proliferation. Furthermore the proteomics revealed significant dysregulation of proteins involved in these processes with α-synuclein-expressing cells having lower levels of proteins involved in phagocytosis, but elevated levels of proteins involved in the response to and uptake of bacteria into pathogen-containing vesicles. The phagocytic vesicle associated proteins that were upregulated were mainly related to membrane fusion and protein transport. While the down regulated proteins were associated mainly with actin binding and the cytoskeleton. In relation to response to bacterium, proteins involved in vesicle transport were upregulated (rab1A, rab5A, rasG, sasA, and vatC). The interaction of tau with α-synuclein in these same cortical regions can explain the ability of tau to reverse the inhibition of phagocytosis by α-synuclein, when tau on its own has no effect on this phenotype, despite also downregulating expression of proteins involved in this pathway.

The colocalisation of tau and α-synuclein at the cortex has been observed in cellular models where tau and α-synuclein have both been found to interact with the plasma membrane (Brandt et al., 1995; Nakamura et al., 2001) and α-synuclein stimulated phosphorylation of tau has been seen to occur here too (Jensen et al., 1999). Esposito et al. (2007) proposed a membrane-bound functional complex with tau and α-synuclein that may involve the actin cytoskeleton. In a Chinese hamster ovary cell line, α-synuclein was found interacting with actin at the plasma membrane and the colocalisation of tau and α-synuclein was highest at the cell periphery. The colocalisation of tau and α-synuclein has been seen in autopsied brain sections of patients with AD (Hamilton, 2000; Arai et al., 2001), cellular models (Badiola et al., 2011) and in LB from patients with LBD (Ishizawa et al., 2003). *D. discoideum* is an accepted model for investigating microtubule dynamics and interactions with microtubule associated proteins (MAPs) and is the best understood model for actin dynamics and function in eukaryotic cells (Eichinger et al., 1999; Noegel and Schleicher, 2000; Gerisch, 2009). An interaction between actin and MTs at the cell cortex has been established (Hestermann et al., 2002) and *D. discoideum* has many homologues of the mammalian MAPs (Graf et al., 2000; Rehberg and Gräf, 2002; Rehberg et al., 2005; Koch et al., 2006). The results presented here indicate an interaction of tau, tubulin and α-synuclein which could be further investigated in the future exploiting the well-established cytoskeletal genetics and molecular biology of the *D. discoideum* model.

There has been much evidence to suggest that mitochondrial dysfunction is involved in neurodegenerative diseases (Lin and Beal, 2006) and both tau and α-synuclein have been implicated. As *D. discoideum* has been well characterised as a model for mitochondrial disease exhibiting a clear set of phenotypes, we analysed the phenotypes of strains expressing tau or α-synuclein or both to determine which might be shared with mitochondrially diseased strains. The α-synuclein-mediated phenotypes and those caused by mitochondrial dysfunction have been compared previously and shown to be distinct (Fernando et al., 2020). Whereas impaired mitochondrial function caused greater intracellular *L. pneumophila* proliferation as well as defects in phototaxis, thermotaxis, growth and development, but did not impair phagocytosis or pinocytosis. Despite similarities in some of these phenotypes, the expression of wild type α-synuclein differed in that it impaired phagocytosis and *L. pneumophila* proliferation, while having no significant effect on growth in liquid, phototaxis, thermotaxis, or fruiting body morphology. In fact, direct assay of mitochondrial function showed that α-synuclein expression did not impair but enhanced mitochondrial respiration. The overall pattern of phenotypes suggested that α-synuclein cytotoxicity lies not in mitochondrial defects but in its impairment of specific endocytic pathways (Fernando et al., 2020).

In this work we report for the first time that tau expression increases *L. pneumophila* susceptibility, has no significant effect on phagocytosis or pinocytosis, impairs growth in liquid but not on bacterial lawns, and causes moderate phototaxis and thermotaxis defects as well as aberrant fruiting bodies with shorter, thicker stalks. This pattern of phenotypic outcomes is very distinct to those caused by α-synuclein expression and indicates that tau and α-synuclein cause different cytotoxic effects in *D. discoideum*. In fact the phenotypic consequences of tau expression are reminiscent of mitochondrial disease phenotypes, with the exception of the normal plaque expansion rates in tau-expressing strains. When mitochondrial respiratory function was measured using Seahorse respirometry, we found an isolated defect in ATP synthesis by complex V, accompanied by an elevation of the mitochondrial “proton leak” (the use of the mitochondrial proton gradient to drive diverse mitochondrial transport processes other than ATP synthesis). Were it not for the normal growth on bacterial lawns, the cytopathological effects of tau expression in *D. discoideum* could thus be attributed entirely to this mitochondrial defect. This raises the question of what other mechanisms might be involved.

The phenotypic abnormalities caused by tau all involved the cytoskeleton and this corresponds with the downregulation of actin cytoskeletal proteins found in the proteomic analysis. Tau impaired axenic growth with a slower generation time in liquid compared to the WT, but this was not mediated by a pinocytosis defect and there was no significant downregulation of proteins associated with the macropinocytic cup. This suggests that other pathways mediating cell proliferation could be causing the defect. This could be a defect in cytokinesis as proteins involved in this process were down-regulated. *Dictyostelium* cytokinesis during growth in suspension in liquid medium depends entirely on the actomyosin cytoskeleton (Zang et al., 1997; Bosgraaf and van Haastert, 2006), whereas on surfaces it can take place by a different mechanism (De Lozanne and Spudich, 1987; Knecht and Loomis, 1987). In the tau transformant the Rho-related protein racE was downregulated. Larochelle et al. (1996) showed that racE was necessary for cytokinesis as *D. discoideum* mutants that did not express racE did not grow in suspension due to a cytokinesis defect. The same group found that racE cells containing an expression vector for racE were able to produce racE to wildtype levels and had no defect in cytokinesis and growth rates in suspension (Larochelle et al., 1997). A study detailing the effect of an *abiA* null mutant (part of the SCAR/WAVE complex that drives actin polymerisation) in *D. discoideum* showed that the axenic growth defect was due to a defect in cytokinesis (Pollitt and Insall, 2008). Tau-expressing strains also exhibited an increased susceptibility to intracellular *L. pneumophila* proliferation, which again corresponded with proteomics data that indicated proteins responsible for the cellular response to bacteria were down-regulated.

In view of the distinctive and sometimes opposite phenotypic outcomes of expressing tau and α-synuclein in *D. discoideum*, the question arises as to what happens in cotransformants expressing both proteins. We showed here that the cotransformants displayed a third distinct pattern of phenotypes, with the presence of the second protein either exacerbating (phototaxis, fruiting body morphology), reversing (phagocytosis, growth on plates, mitochondrial respiratory function, *L. pneumophila* proliferation), or having no significant impact (growth in liquid) on defects caused by the other. This indicates clear functional interactions of the two proteins in several phenotypic pathways and in some cases a synergistic effect. There is similar evidence to suggest an interaction and synergistic cytotoxicity of these two proteins in other model systems coexpressing tau and α-synuclein. In cellular models Badiola et al. (2011) found that tau and α-synuclein colocalised in primary neuronal cultures and the overexpression of tau lead to enhanced α-synuclein cytotoxicity. In a *Drosophila* model of PD, Roy and Jackson (2014) misexpressed tau and α-synuclein singly and in combination. They found that the expression of α-synuclein produced no phenotype associated with the eye, while the expression of tau caused the rough eye phenotype with smaller eyes and the combined expression of the two proteins resulted in a more severe phenotype. This accords with our finding that α-synuclein exacerbates tau-mediated defects in phototaxis and fruiting body morphology. Similarly in yeast, tau exacerbated the growth defects caused by α-synuclein (tau alone produced no defect) (Ciaccioli et al., 2013).

Although our results support a cytotoxic interaction between the two proteins in some phenotypes, we also found that in relation to other phenotypes the two proteins exerted opposing actions. Thus the significantly impaired growth on bacterial lawns and the phagocytosis defect observed in the α-synuclein strains were reversed in the cotransformants. In the case of intracellular *L. pneumophila* proliferation, tau not only reversed the reduction caused by α-synuclein, but increased the proliferation of the pathogen to the same elevated levels as observed when tau was expressed singly. There is evidence that tau affects neuronal phagocytosis (Xie et al., 2019) and α-synuclein affects synaptic endocytosis (Lautenschläger et al., 2017), but the effect of combined expression on endocytic pathways has not been previously reported.

Another of the phenotypes in which coexpression of tau and α-synuclein had opposing effects was in mitochondrial respiratory function. Previously we showed that α-synuclein did not cause an impairment in mitochondrial function but instead it increased respiratory activity coordinately in all components measured (Fernando et al., 2020). This result was consistent with increases in mitochondrial respiration seen in lymphoblast cell lines made from iPD patients (Annesley et al., 2016), fibroblasts from iPD patients (Haylett et al., 2016) and when neuroblastoma cells were seeded with α-synuclein fibrils (Ugalde et al., 2020). In this work we found that tau expression caused a significant decrease in the OCR dedicated to ATP synthesis thereby revealing a specific defect in Complex V. A decrease in ATP production has been seen in neuronal cultures overexpressing tau (Li et al., 2016) and this was accompanied by decreases in Complex I activity and in the ratio of ATP/ADP. Here we found no Complex I defect caused by tau in *D. discoideum.* The combined expression of tau and α-synuclein returned mitochondrial respiration to normal, reversing the elevated mitochondrial respiration caused by α-synuclein as well as the Complex V defect and elevated proton leak caused by tau. Altered mitochondrial function is associated with many neurodegenerative diseases and this result highlights that it may occur through different mechanisms and the importance of looking at neuronal protein-protein interactions to advance our understanding of the part played by mitochondria in neurodegeneration. This emphasises the usefulness of using a simple model to study complex interactions and processes.

Many of the defective phenotypes observed in the tau-expressing strains were in line with mitochondrial dysfunction and previously this has been shown to be due to the chronic activation of AMPK (Bokko et al., 2007; Francione et al., 2009). To determine if there was a functional relationship between tau and AMPK in *D. discoideum*, we created cotransformants which expressed tau or tau and α-synuclein and antisense inhibited AMPK. As phototaxis is a signature defect of impaired mitochondrial function we investigated whether this phenotype was mediated by increased AMPK activity. The antisense inhibition of AMPK in tau strains and strains expressing both tau and α-synuclein resulted in a rescue of the phototaxis defect, suggesting that AMPK mediates the phototaxis defect caused by tau and exacerbated by α-synuclein coexpression. This suggests that AMPK may have been upregulated in the transformants, however, we could not measure this directly as AMPK could not be detected in any of the transformants or wild type strains. In neurons containing tau pathology in AD and many other tauopathies, AMPK levels and degree of activation (phosphorylation) are elevated (Vingtdeux et al., 2011). In cell culture a physical interaction between AMPK and tau was established and overexpression of AMPK increased tau toxicity and phosphorylation (Galasso et al., 2017). Interestingly, the inhibition of AMPK might serve as neuronal protection in neurodegenerative diseases (McCullough et al., 2005). Models of motor neuron disease and amylotrophic lateral sclerosis found benefits of downregulating AMPK (Lim et al., 2012). In *Drosophila* expressing tau downregulation of the AMPKα subunit partially rescued the tau rough eye phenotype. In future studies, determining if AMPK knockdown can rescue the other tau-mediated phenotypes would be beneficial, as would investigating whether AMPK phosphorylates tau in *D. discoideum.*

Proteomics analysis revealed that there were a number of proteins up- and down-regulated in these strains. In all strains more proteins were down-regulated than up-regulated, and the combined effect of tau and α-synuclein coexpression dysregulated the expression of more proteins than did expression of either protein on its own. The pattern of dysregulation caused by tau and α-synuclein expression were quite distinct from each other. Tau significantly up-regulated protein degradation and turnover, possibly in response to the defect in ATP synthesis, while α-synuclein up-regulated proteins involved in the response to bacterium corresponding with decreased *Legionella* susceptibility possibly as a result of a more efficient endolysosomal pathway. All strains exhibited a down-regulation of cytoskeletal proteins, which was exacerbated in the cotransformants. Interestingly here, the cotransformants displayed unique downregulation in some aspects of the cytoskeleton relating to cell motility, polarity, morphogenesis, and filopodium assembly corresponding with the more severe phototaxis defect. Both tau and α-synuclein down-regulated proteins involved in lipid and steroid metabolism, which are dysregulated in diseases like AD and PD. However, these pathways were not affected in the cotransformants, again consistent with a functional interaction between these two proteins that is sometimes beneficial rather than cytotoxic.

## Conclusion

Our results show that *D. discoideum* can be a useful model to study the biological functions of tau and the interactions with other neurodegeneration-associated proteins. Whilst neurodegenerative disease like Alzheimer’s and Parkinson’s is characterised by death or dysfunction of neurons research is accumulating to suggest that the underlying disease mechanisms are likely to be more systemic occurring in many more cell types and not just isolated to neurons. We believe that by understanding the underlying molecular pathology that is shared by all cell types then we can understand why particular neurons or parts of the brain are selectively affected in these disorders. Here we used an organism which does not contain neurons, or a brain but it does contain many conserved pathways and proteins with humans and has been shown to be a good model for studying mitochondrial function, cell division, growth, endocytosis, autophagy and intracellular signalling all of which have been implicated in neurodegenerative disease. Here we expressed tau alone and in combination with α-synuclein to investigate the cytotoxic effects and interactions between the proteins in this simple model system. The results showed that tau and α-synuclein have different subcellular distributions but they colocalise in the cortical regions of the cell. They affect different pathways and phenotypes when expressed singly and, depending on the pathway and phenotype, these effects can be enhanced or reversed by the expression of both proteins at once. Thus, the *D. discoideum* model has revealed that the α-synuclein/tau interaction is clear but more complex than a simple synergistic cytotoxicity. These complexities are worthy of further investigation in models like *D. discoideum* in which they can be studied without concerns about the possible effects of endogenous orthologues.

## Data Availability Statement

The datasets presented in this study can be found in online repositories. The name of the repository and accession number can be found below: MassIVE, https://massive.ucsd.edu/ProteoSAFe/static/massive.jsp, MSV000087848.

## Author Contributions

KM made the tau construct and tau strains including cotransformants, performed all of the experiments for the tau and cotransformant strains, analysed the data, and prepared, reviewed, and edited the original manuscript. SF made the α-syn constructs and strains and performed most of the phenotypic experiments with these strains. PF and SA conceptualised the project, supervised KM, advised on data analysis, and reviewed/edited the manuscript. All authors have read and agreed to the published version of the manuscript.

## Conflict of Interest

The authors declare that the research was conducted in the absence of any commercial or financial relationships that could be construed as a potential conflict of interest.

## Publisher’s Note

All claims expressed in this article are solely those of the authors and do not necessarily represent those of their affiliated organizations, or those of the publisher, the editors and the reviewers. Any product that may be evaluated in this article, or claim that may be made by its manufacturer, is not guaranteed or endorsed by the publisher.

## Supplementary Material

Supplementary Table 1 Tau up-regulated proteins.

Supplementary Table 2 Tau down-regulated proteins.

Supplementary Table 3 α-Synuclein up-regulated proteins.

Supplementary Table 4 α-Synuclein down-regulated proteins.

Supplementary Table 5 Cotransformant up-regulated proteins.

Supplementary Table 6 Cotransformant down-regulated proteins.
