# Supplementary material for: Interactions and Cytotoxicity of Human Neurodegeneration- Associated Proteins Tau and α-Synuclein in the Simple Model Dictyostelium discoideum
Source: Front Cell Dev Biol. 2021 Sep 6;9:741662. doi: 10.3389/fcell.2021.741662 (PMC8450459; doi:10.3389/fcell.2021.741662)
Supplement: Supplementary Table 1 — Tau up-regulated proteins. [file Table_1.DOCX]

| **Up-regulated processes** |
| --- |
| Proteyolysis |
| Positive regulation of RNA polymerase II transcription preinitiation complex |
| Translation |
| tRNA aminoacylation for protein translation |
| Other |
| Disconnected node |

**Supplemental Information**

**Protein annotations taken from STRING.**

**Table S1: Tau up-regulated proteins**

*Proteins with multiple colours involved in more than one process.

| **#node** | **Identifier** | **Annotation** |
| --- | --- | --- |
| DDB0168140 | DDB0168140 | annotation not available |
| DDB0190682 | DDB0233387 | annotation not available |
| DDB0191832 | DDB0233013 | annotation not available |
| DDB0192224 | DDB0192224 | annotation not available |
| DDB0204927 | DDB0204927 | annotation not available |
| DDB0217073 | DDB0234178 | annotation not available |
| DDB0218284 | DDB0218284 | annotation not available |
| DDB0230005 | DDB0230005 | CBS domain-containing protein DDB_G0289609 |
| DDB0230064 | DDB0230064 | annotation not available |
| DDB0233715 | DDB0233715 | annotation not available |
| argS1 | DDB0231324 | Probable arginine--tRNA ligase, cytoplasmic; Forms part of a macromolecular complex that catalyzes the attachment of specific amino acids to cognate tRNAs during protein synthesis |
| asns | DDB0230140 | Probable asparagine synthetase [glutamine-hydrolyzing] |
| aspS1 | DDB0231308 | Aspartyl-tRNA synthetase, cytoplasmic 1; Belongs to the class-II aminoacyl-tRNA synthetase family. Type 2 subfamily |
| cinB | DDB0220110 | Vegetative-specific protein H5; Belongs to the 'GDXG' lipolytic enzyme family |
| eif3L | DDB0233946 | Eukaryotic translation initiation factor 3 subunit L; Component of the eukaryotic translation initiation factor 3 (eIF-3) complex, which is involved in protein synthesis of a specialized repertoire of mRNAs and, together with other initiation factors, stimulates binding of mRNA and methionyl-tRNAi to the 40S ribosome. The eIF-3 complex specifically targets and initiates translation of a subset of mRNAs involved in cell proliferation |
| erf3 | DDB0214990 | Eukaryotic peptide chain release factor GTP-binding subunit; Involved in translation termination. Stimulates the activity of erf1. Binds guanine nucleotides (By similarity) |
| fbl | DDB0267046 | rRNA 2'-O-methyltransferase fibrillarin; S-adenosyl-L-methionine-dependent methyltransferase that has the ability to methylate both RNAs and proteins. Involved in pre-rRNA processing. Utilizes the methyl donor S-adenosyl-L- methionine to catalyze the site-specific 2'-hydroxyl methylation of ribose moieties in pre-ribosomal RNA. Site specificity is provided by a guide RNA that base pairs with the substrate. Methylation occurs at a characteristic distance from the sequence involved in base pairing with the guide RNA. Also acts as a protein methyltransferase by mediating methylation of 'G [...] |
| fpa2 | DDB0266780 | SCF ubiquitin ligase complex protein SKP1bSCF ubiquitin ligase complex protein SKP1b(4-162)SCF ubiquitin ligase complex protein SKP1b(6-162) |
| g6pd-1 | DDB0238739 | Glucose-6-phosphate 1-dehydrogenase; Catalyzes the rate-limiting step of the oxidative pentose-phosphate pathway, which represents a route for the dissimilation of carbohydrates besides glycolysis. The main function of this enzyme is to provide reducing power (NADPH) and pentose phosphates for fatty acid and nucleic acid synthesis (By similarity) |
| gluS | DDB0231321 | Probable glutamate--tRNA ligase, cytoplasmic; Catalyzes the attachment of glutamate to tRNA(Glu) in a two-step reaction: glutamate is first activated by ATP to form Glu-AMP and then transferred to the acceptor end of tRNA(Glu); Belongs to the class-I aminoacyl-tRNA synthetase family. Glutamate--tRNA ligase type 2 subfamily |
| gpaD | DDB0216411 | Guanine nucleotide-binding protein alpha-4 subunit; Guanine nucleotide-binding proteins (G proteins) are involved as modulators or transducers in various transmembrane signaling systems. G alpha-4 plays a role in morphogenesis of the multicellular structure |
| hisS | DDB0231332 | Histidine--tRNA ligase, cytoplasmic; Histidyl-tRNA synthetase, cytoplasmic; Belongs to the class-II aminoacyl-tRNA synthetase family |
| hspC | DDB0185048 | 32 kDa heat shock protein |
| leuS | DDB0231253 | Leucine--tRNA ligase, cytoplasmic; Leucyl-tRNA synthetase, cytoplasmic; Belongs to the class-I aminoacyl-tRNA synthetase family |
| ndkB | DDB0238334 | Nucleoside diphosphate kinase, cytosolic; Major role in the synthesis of nucleoside triphosphates other than ATP |
| nop5 | DDB0305289 | Nucleolar protein 58; Required for 60S ribosomal subunit biogenesis |
| nploc4 | DDB0233722 | Nuclear protein localization protein 4 homolog; May be part of a complex that binds ubiquitinated proteins and that is necessary for the export of misfolded proteins from the ER to the cytoplasm, where they are degraded by the proteasome |
| ppkA | DDB0216190 | Polyphosphate kinase; Catalyzes the reversible transfer of the terminal phosphate of ATP to form a long-chain polyphosphate (polyP). Produces polyP in a broad range of chain lengths (50-300 Pi residues). Involved in development (growth and fruiting body formation), sporulation, phagocytosis, cell division and the late stages of cytokinesis |
| psmC1 | DDB0232964 | 26S proteasome regulatory subunit 4 homolog; The 26S proteasome is involved in the ATP-dependent degradation of ubiquitinated proteins. The regulatory (or ATPase) complex confers ATP dependency and substrate specificity to the 26S complex (By similarity). Plays an important role in regulating both growth and multicellular development |
| psmC2 | DDB0232966 | 26S proteasome regulatory subunit 7; The 26S proteasome is involved in the ATP-dependent degradation of ubiquitinated proteins. The regulatory (or ATPase) complex confers ATP dependency and substrate specificity to the 26S complex (By similarity) |
| psmC3 | DDB0232967 | 26S proteasome regulatory subunit 6A homolog; The 26S proteasome is involved in the ATP-dependent degradation of ubiquitinated proteins. The regulatory (or ATPase) complex confers ATP dependency and substrate specificity to the 26S complex (By similarity) |
| psmC6 | DDB0232968 | 26S proteasome regulatory subunit 10B; The 26S proteasome is involved in the ATP-dependent degradation of ubiquitinated proteins. The regulatory (or ATPase) complex confers ATP dependency and substrate specificity to the 26S complex (By similarity) |
| psmD14 | DDB0191298 | 26S proteasome non-ATPase regulatory subunit 14; Metalloprotease component of the 26S proteasome that specifically cleaves 'Lys-63'-linked polyubiquitin chains. The 26S proteasome is involved in the ATP-dependent degradation of ubiquitinated proteins. The function of the 'Lys-63'-specific deubiquitination of the proteasome is unclear (By similarity) |
| psmD4 | DDB0232981 | 26S proteasome non-ATPase regulatory subunit 4; Binds and presumably selects ubiquitin-conjugates for destruction |
| psmD7 | DDB0232987 | 26S proteasome non-ATPase regulatory subunit 7; Acts as a regulatory subunit of the 26S proteasome which is involved in the ATP-dependent degradation of ubiquitinated proteins |
| pyrK | DDB0191367 | UMP-CMP kinase; Catalyzes the phosphorylation of pyrimidine nucleoside monophosphates at the expense of ATP. Plays an important role in de novo pyrimidine nucleotide biosynthesis. Has preference for UMP and CMP as phosphate acceptors; Belongs to the adenylate kinase family. UMP-CMP kinase subfamily |
| rbrA | DDB0191418 | Probable E3 ubiquitin-protein ligase rbrA; Might act as an E3 ubiquitin-protein ligase. Appears to be required for normal cell-type proportioning and cell sorting during multicellular development. In addition to being necessary for a normal percentage of prestalk cells and the organization of the slug, rbrA is also necessary for spore cell viability |
| tbpB | DDB0191435 | 26S proteasome regulatory subunit 6B homolog; The 26S proteasome is involved in the ATP-dependent degradation of ubiquitinated proteins. The regulatory (or ATPase) complex confers ATP dependency and substrate specificity to the 26S complex (By similarity) |
| tbpC | DDB0216230 | 26S proteasome regulatory subunit 8; The 26S proteasome is involved in the ATP-dependent degradation of ubiquitinated proteins. The regulatory (or ATPase) complex confers ATP dependency and substrate specificity to the 26S complex (By similarity) |
| udpB | DDB0230170 | annotation not available |
| vatB | DDB0185207 | V-type proton ATPase subunit B; Vacuolar ATPase is responsible for acidifying a variety of intracellular compartments in eukaryotic cells. The B subunit is non-catalytic but combines with other subunits to form the catalytic complex. V-ATPase is responsible for energizing electrophoretic K(+)/2H(+) antiport by generating a transmembrane voltage of more than 200 mV (By similarity) |
| vatE | DDB0185070 | V-type proton ATPase subunit E; Subunit of the peripheral V1 complex of vacuolar ATPase essential for assembly or catalytic function. V-ATPase is responsible for acidifying a variety of intracellular compartments in eukaryotic cells (By similarity) |

| **Down-regulated processes** |
| --- |
| Actin cytoskeleton |
| Steroid biosynthetic process |
| Oxidation/reduction |
| Lipid metabolic process |
| Other |
| Disconnected node |

**Table S2: Tau down-regulated proteins**

*Proteins with multiple colours involved in more than one process.

| **#node** | **Identifier** | **Annotation** |
| --- | --- | --- |
| DDB0167402 | DDB0238277 | Uncharacterized protein; Belongs to the short-chain dehydrogenases/reductases (SDR) family |
| DDB0168996 | DDB0168996 | annotation not available |
| DDB0169413 | DDB0169413 | annotation not available |
| DDB0169464 | DDB0234149 | annotation not available |
| DDB0169506 | DDB0231973 | annotation not available |
| DDB0185931 | DDB0185931 | annotation not available |
| DDB0186109 | DDB0186109 | annotation not available |
| DDB0187592 | DDB0187592 | annotation not available |
| DDB0188084 | DDB0234168 | annotation not available |
| DDB0188715 | DDB0234195 | annotation not available |
| DDB0188843 | DDB0188843 | annotation not available |
| DDB0191909 | DDB0191909 | Uncharacterized protein |
| DDB0202574 | DDB0202574 | annotation not available |
| DDB0205386 | DDB0235361 | annotation not available |
| DDB0205662 | DDB0205662 | annotation not available |
| DDB0219884 | DDB0233782 | annotation not available |
| DDB0231475 | DDB0231475 | Aldehyde dehydrogenase; Belongs to the aldehyde dehydrogenase family |
| DDB0231504 | DDB0231504 | Putative aldehyde dehydrogenase family 7 member A1 homolog; Belongs to the aldehyde dehydrogenase family |
| DDB0232204 | DDB0232204 | Aminotransferase class-III; Belongs to the class-III pyridoxal-phosphate-dependent aminotransferase family |
| DDB0233285 | DDB0233285 | PH domain-containing protein DDB_G0274775 |
| DDB0233382 | DDB0233382 | Short-chain dehydrogenase/reductase family protein; Belongs to the short-chain dehydrogenases/reductases (SDR) family |
| DDB0234107 | DDB0234107 | annotation not available |
| DDB0234207 | DDB0234207 | annotation not available |
| DDB0237843 | DDB0237843 | annotation not available |
| DDB0267057 | DDB0267057 | Thimet-like oligopeptidase |
| abcG9 | DDB0214893 | ABC transporter G family member 9 |
| abpA | DDB0191133 | Alpha-actinin A; F-actin cross-linking protein which is thought to anchor actin to a variety of intracellular structures. This is a bundling protein. Increases the actin-stimulated ATPase activity of myosin. Involved in vegetative cell growth, phagocytosis, motility and development, probably through stabilization of the actin network in the cortical cytoskeleton |
| acly | DDB0235360 | Probable ATP-citrate synthase; ATP-citrate synthase is the primary enzyme responsible for the synthesis of cytosolic acetyl-CoA in many tissues; In the N-terminal section; belongs to the succinate/malate CoA ligase beta subunit family |
| acpA | DDB0191202 | F-actin-capping protein subunit beta; F-actin-capping proteins bind in a Ca(2+)-independent manner to the fast growing ends of actin filaments (barbed end) thereby blocking the exchange of subunits at these ends. Unlike other capping proteins (such as gelsolin and severin), these proteins do not sever actin filaments |
| act | DDB0219936 | Actin-related protein 3; Functions as ATP-binding component of the Arp2/3 complex which is involved in regulation of actin polymerization and together with an activating nucleation-promoting factor (NPF) mediates the formation of branched actin networks. Seems to contact the pointed end of the daughter actin filament. The Arp2/3 complex is involved in organizing the actin system in cell motility and chemotaxis, in phagocytosis and macropinocytosis, at late steps of endosome processing, and in mitosis. In concert with a group of other proteins, the Arp2/3 complex plays a general role in [...] |
| adprt1A | DDB0214818 | Poly [ADP-ribose] polymerase |
| aip1 | DDB0214916 | Actin-interacting protein 1; Implicated in both actin filament depolymerization and polymerization. May enhance chemotaxis by promoting cofilin- dependent actin assembly at cell leading edges |
| alrA | DDB0215363 | Aldose reductase A; Catalyzes the NADPH-dependent reduction of a wide variety of carbonyl-containing compounds to their corresponding alcohols with a broad range of catalytic efficiencies (By similarity). Probably affects several metabolic pathways in addition to converting glucose to sorbitol. Affects group size |
| amyA | DDB0214924 | Putative alpha-amylase; AmyA |
| ancA | DDB0201558 | Mitochondrial substrate carrier family protein ancA; Mitochondrial solute carriers shuttle metabolites, nucleotides, and cofactors through the mitochondrial inner membrane. Catalyzes the exchange of ADP and ATP across the mitochondrial inner membrane |
| arcA | DDB0214932 | Actin-related protein 2/3 complex subunit 1; Functions as component of the Arp2/3 complex which is involved in regulation of actin polymerization and together with an activating nucleation-promoting factor (NPF) mediates the formation of branched actin networks. Seems to contact the pointed end of the daughter actin filament. The Arp2/3 complex is involved in organizing the actin system in cell motility and chemotaxis, in phagocytosis and macropinocytosis, at late steps of endosome processing, and in mitosis. In concert with a group of other proteins, the Arp2/3 complex plays a general [...] |
| arcB | DDB0214935 | Actin-related protein 2/3 complex subunit 2; Functions as component of the Arp2/3 complex which is involved in regulation of actin polymerization and together with an activating nucleation-promoting factor (NPF) mediates the formation of branched actin networks. Seems to contact the pointed end of the daughter actin filament. The Arp2/3 complex is involved in organizing the actin system in cell motility and chemotaxis, in phagocytosis and macropinocytosis, at late steps of endosome processing, and in mitosis. In concert with a group of other proteins, the Arp2/3 complex plays a general [...] |
| arcC | DDB0201632 | Actin-related protein 2/3 complex subunit 3; Functions as component of the Arp2/3 complex which is involved in regulation of actin polymerization and together with an activating nucleation-promoting factor (NPF) mediates the formation of branched actin networks. Seems to contact the pointed end of the daughter actin filament. The Arp2/3 complex is involved in organizing the actin system in cell motility and chemotaxis, in phagocytosis and macropinocytosis, at late steps of endosome processing, and in mitosis. In concert with a group of other proteins, the Arp2/3 complex plays a general [...] |
| cnrI | DDB0229864 | Putative countin receptor Cnr9; SET domain-containing protein |
| coaA | DDB0215369 | Coactosin; Binds to F-actin in a calcium independent manner. Binds to the filaments along their length |
| cofA | DDB0214987 | Cofilin-1A; Controls reversibly actin polymerization and depolymerization in a pH-sensitive manner. It has the ability to bind G- and F-actin in a 1:1 ratio of cofilin to actin. It is the major component of intranuclear and cytoplasmic actin rods (By similarity) |
| corA | DDB0191115 | Coronin-A; Required for normal motility. Participates in cytokinesis |
| cyb5r1 | DDB0266821 | NADH-cytochrome b5 reductase 1; Electron donor reductase for cytochrome b5. The cytochrome b5/NADH cytochrome b5 reductase electron transfer system supports the catalytic activity of several sterol biosynthetic enzymes (By similarity) |
| cyp51 | DDB0232962 | Probable lanosterol 14-alpha demethylase; Catalyzes C14-demethylation of lanosterol which is critical for ergosterol biosynthesis. It transforms lanosterol into 4,4'-dimethyl cholesta-8,14,24-triene-3-beta-ol (By similarity) |
| cyp524A1 | DDB0233032 | Probable cytochrome P450 524A1 |
| dpm1 | DDB0231708 | Dolichol-phosphate mannosyltransferase subunit 1; Transfers mannose from GDP-mannose to dolichol monophosphate to form dolichol phosphate mannose (Dol-P-Man) which is the mannosyl donor in pathways leading to N-glycosylation, glycosyl phosphatidylinositol membrane anchoring, and O- mannosylation of proteins; catalytic subunit of the dolichol- phosphate mannose (DPM) synthase complex |
| eapA | DDB0191146 | Alkyldihydroxyacetonephosphate synthase; Catalyzes the exchange of an acyl for a long-chain alkyl group and the formation of the ether bond in the biosynthesis of ether phospholipids |
| empC | DDB0215345 | Emp24/gp25L/p24 family proteinPutative uncharacterized protein empC |
| erg24 | DDB0232079 | Delta(14)-sterol reductase; Reduces the C14=C15 double bond of 4,4-dimethyl- cholesta-8,14,24-trienol to produce 4,4-dimethyl-cholesta-8,24- dienol; Belongs to the ERG4/ERG24 family |
| fcsA | DDB0191105 | Fatty acyl-CoA synthetase A; Long chain fatty acid acyl-CoA synthetases catalyze the formation of a thiester bond between a free fatty acid and coenzyme A during fatty acid metabolic process. May mediate fatty acid retrieval from the lumen of endosomes into the cytoplasm; Belongs to the ATP-dependent AMP-binding enzyme family |
| fhbA | DDB0191099 | Flavohemoprotein A; Is involved in NO detoxification in an aerobic process, termed nitric oxide dioxygenase (NOD) reaction that utilizes O(2) and NAD(P)H to convert NO to nitrate, which protects the cell from various noxious nitrogen compounds. Therefore, plays a central role in the inducible response to nitrosative stress; In the C-terminal section; belongs to the flavoprotein pyridine nucleotide cytochrome reductase family |
| fimA | DDB0214994 | Fimbrin; Binds to actin |
| fps | DDB0215017 | Farnesyl diphosphate synthase; Key enzyme in isoprenoid biosynthesis which catalyzes the formation of farnesyl diphosphate (FPP), a sterol precursor. Involved in the inhibition of cell growth; Belongs to the FPP/GGPP synthase family |
| glgB | DDB0214943 | 1,4-alpha-glucan-branching enzyme |
| gloB2 | DDB0230991 | Glyoxylase B2 |
| glud2 | DDB0233691 | Glutamate dehydrogenase 2; Belongs to the Glu/Leu/Phe/Val dehydrogenases family |
| haao | DDB0231359 | 3-hydroxyanthranilate 3,4-dioxygenase; Catalyzes the oxidative ring opening of 3- hydroxyanthranilate to 2-amino-3-carboxymuconate semialdehyde, which spontaneously cyclizes to quinolinate |
| hemE | DDB0231418 | Uroporphyrinogen decarboxylase; Catalyzes the decarboxylation of four acetate groups of uroporphyrinogen-III to yield coproporphyrinogen-III |
| hexa1 | DDB0191256 | Beta-hexosaminidase subunit A1; Responsible for the degradation of GM2 gangliosides, and a variety of other molecules containing terminal N-acetyl hexosamines. This enzyme plays a role during the slug stage of development in the maintenance of pseudoplasmodia of normal size; Belongs to the glycosyl hydrolase 20 family |
| hgd | DDB0191461 | Homogentisate 1,2-dioxygenase |
| hgsA | DDB0219924 | Hydroxymethylglutaryl-CoA synthase A; Condenses acetyl-CoA with acetoacetyl-CoA to form HMG- CoA, which is the substrate for HMG-CoA reductase |
| idhC | DDB0231401 | Isocitrate dehydrogenase [NADP] cytoplasmic; Belongs to the isocitrate and isopropylmalate dehydrogenases family |
| maoA | DDB0231707 | Probable flavin-containing monoamine oxidase A; Belongs to the flavin monoamine oxidase family |
| mppB | DDB0231799 | Mitochondrial-processing peptidase subunit beta; The mitochondrial processing protease (MPP-I) cleaves presequences from mitochondrial protein precursors. Most MPP-I cleavage sites follow an arginine at position -2. mppB is the catalytic subunit of the heterodimeric metallo-endopeptidase. Mitochondrial processing peptidase plays an essential role in mitochondrial biogenesis |
| mvd | DDB0252847 | Diphosphomevalonate decarboxylase; Performs the first committed step in the biosynthesis of isoprenes; Belongs to the diphosphomevalonate decarboxylase family |
| nedd8 | DDB0238041 | NEDD8; Ubiquitin-like protein which plays an important role in cell cycle control, embryogenesis and neurogenesis. Covalent attachment to its substrates requires prior activation by the E1 complex ube1c/uba3-ula1 and linkage to the E2 enzyme ube2m/ubc12. Attachment of nedd8 to cullins activates their associated E3 ubiquitin ligase activity, and thus promotes polyubiquitination and proteasomal degradation of cyclins and other regulatory proteins (By similarity) |
| nxnA | DDB0232009 | Annexin A7; Calcium/phospholipid-binding protein which promotes membrane fusion and is involved in exocytosis |
| omt6 | DDB0229909 | Probable caffeoyl-CoA O-methyltransferase 2 |
| ost1 | DDB0233146 | Dolichyl-diphosphooligosaccharide--protein glycosyltransferase subunit 1; Essential subunit of the N-oligosaccharyl transferase (OST) complex which catalyzes the transfer of a high mannose oligosaccharide from a lipid-linked oligosaccharide donor to an asparagine residue within an Asn-X-Ser/Thr consensus motif in nascent polypeptide chains; Belongs to the OST1 family |
| pgmA | DDB0191348 | Phosphoglucomutase-1; This enzyme participates in both the breakdown and synthesis of glucose; Belongs to the phosphohexose mutase family |
| pkiA | DDB0216234 | Protein pkiA |
| pksB | DDB0214951 | PksBShort-chain dehydrogenase/reductase family protein; Belongs to the short-chain dehydrogenases/reductases (SDR) family |
| ponA | DDB0215380 | Ponticulin; Binds F-actin and nucleates actin assembly. Major high affinity link between the plasma membrane and the cortical actin network |
| prlA | DDB0232955 | Proliferation-associated protein A; Belongs to the peptidase M24 family |
| proA | DDB0191178 | Profilin-1; Binds to actin and affects the structure of the cytoskeleton. At high concentrations, profilin prevents the polymerization of actin, whereas it enhances it at low concentrations. By binding to PIP2, it inhibits the formation of IP3 and DG |
| proB | DDB0191249 | Profilin-2; Binds to actin and affects the structure of the cytoskeleton. At high concentrations, profilin prevents the polymerization of actin, whereas it enhances it at low concentrations. By binding to PIP2, it inhibits the formation of IP3 and DG |
| psaB | DDB0231240 | annotation not available |
| purD | DDB0230084 | Bifunctional purine biosynthetic protein purDPhosphoribosylamine--glycine ligasePhosphoribosylformylglycinamidine cyclo-ligase; In the N-terminal section; belongs to the GARS family |
| rab7A | DDB0191507 | Ras-related protein Rab-7A; Key regulator in endo-lysosomal trafficking. Governs early-to-late endosomal maturation, microtubule minus-end as well as plus-end directed endosomal migration and positioning, and endosome-lysosome transport through different protein-protein interaction cascades (By similarity). Involved in lipophagy, a cytosolic lipase-independent autophagic pathway (By similarity) |
| rac1A | DDB0214822 | Rho-related protein rac1A; Overexpression promotes the formation of filopodia and membrane ruffles; Belongs to the small GTPase superfamily. Rho family |
| racC | DDB0201659 | Rho-related protein racC; Belongs to the small GTPase superfamily. Rho family |
| racE | DDB0214825 | Rho-related protein racE; Specifically required for cytokinesis; Belongs to the small GTPase superfamily. Rho family |
| rasG | DDB0201663 | Ras-like protein rasG; Ras proteins bind GDP/GTP and possess intrinsic GTPase activity; Belongs to the small GTPase superfamily. Ras family |
| sarA | DDB0229965 | GTP-binding protein Sar1A; Component of the coat protein complex II (COPII) which promotes the formation of transport vesicles from the endoplasmic reticulum (ER). The coat has two main functions, the physical deformation of the endoplasmic reticulum membrane into vesicles and the selection of cargo molecules (By similarity) |
| sasA | DDB0214885 | Ras-related protein Rab-8A; Protein transport. Probably involved in vesicular traffic (By similarity) |
| sec31 | DDB0235185 | Protein transport protein SEC31; Component of the coat protein complex II (COPII) which promotes the formation of transport vesicles from the endoplasmic reticulum (ER). The coat has two main functions, the physical deformation of the endoplasmic reticulum membrane into vesicles and the selection of cargo molecules (By similarity) |
| serA | DDB0230052 | D-3-phosphoglycerate dehydrogenase; Catalyzes the reversible oxidation of 3-phospho-D- glycerate to 3-phosphonooxypyruvate, the first step of the phosphorylated L-serine biosynthesis pathway. Also catalyzes the reversible oxidation of 2-hydroxyglutarate to 2-oxoglutarate; Belongs to the D-isomer specific 2-hydroxyacid dehydrogenase family |
| shmt1 | DDB0230072 | Serine hydroxymethyltransferase 1; Interconversion of serine and glycine |
| smt1 | DDB0237965 | Probable cycloartenol-C-24-methyltransferase 1; Catalyzes the methyl transfer from S-adenosyl-methionine to the C-24 of cycloartenol to form 24-methylene cycloartenol |
| sodC | DDB0232186 | Extracellular superoxide dismutase [Cu-Zn] 3; Protect the extracellular space from toxic effect of reactive oxygen intermediates by converting superoxyde radicals into hydrogen peroxyde and oxygen |
| sqor | DDB0252562 | Sulfide:quinone oxidoreductase, mitochondrial; Catalyzes the oxidation of hydrogen sulfide, with the help of a quinone |
| swp1 | DDB0233147 | Dolichyl-diphosphooligosaccharide--protein glycosyltransferase subunit swp1; Essential subunit of the N-oligosaccharyl transferase (OST) complex which catalyzes the transfer of a high mannose oligosaccharide from a lipid-linked oligosaccharide donor to an asparagine residue within an Asn-X-Ser/Thr consensus motif in nascent polypeptide chains |
| tdo | DDB0231363 | Tryptophan 2,3-dioxygenase; Heme-dependent dioxygenase that catalyzes the oxidative cleavage of the L-tryptophan (L-Trp) pyrrole ring and converts L- tryptophan to N-formyl-L-kynurenine. Catalyzes the oxidative cleavage of the indole moiety |
| thfA | DDB0230118 | Methylenetetrahydrofolate dehydrogenase [NAD(+)]; Catalyzes oxidation of cytoplasmic one-carbon units for purine biosynthesis |
| trxE | DDB0237674 | Putative thioredoxin-5; Participates in various redox reactions through the reversible oxidation of its active center dithiol to a disulfide and catalyzes dithiol-disulfide exchange reactions |
| uox | DDB0231470 | Uricase; Catalyzes the oxidation of uric acid to 5- hydroxyisourate, which is further processed to form (S)-allantoin; Belongs to the uricase family |
| uqcrq | DDB0267111 | Probable cytochrome b-c1 complex subunit 8; This is a component of the ubiquinol-cytochrome c reductase complex (complex III or cytochrome b-c1 complex), which is part of the mitochondrial respiratory chain. This subunit, together with cytochrome b, binds to ubiquinone (By similarity) |

**Table S3: α-synuclein up-regulated proteins**

| **#node** | **Identifier** | **Annotation** |
| --- | --- | --- |
| DDB0191909 | DDB0191909 | Uncharacterized protein |
| DDB0219276 | DDB0219276 | annotation not available |
| DDB0233382 | DDB0233382 | Short-chain dehydrogenase/reductase family protein; Belongs to the short-chain dehydrogenases/reductases (SDR) family |
| DDB0233715 | DDB0233715 | annotation not available |
| arcE | DDB0191138 | Actin-related protein 2/3 complex subunit 5; Functions as component of the Arp2/3 complex which is involved in regulation of actin polymerization and together with an activating nucleation-promoting factor (NPF) mediates the formation of branched actin networks. Seems to contact the pointed end of the daughter actin filament. The Arp2/3 complex is involved in organizing the actin system in cell motility and chemotaxis, in phagocytosis and macropinocytosis, at late steps of endosome processing, and in mitosis. In concert with a group of other proteins, the Arp2/3 complex plays a general [...] |
| cinB | DDB0220110 | Vegetative-specific protein H5; Belongs to the 'GDXG' lipolytic enzyme family |
| coaA | DDB0215369 | Coactosin; Binds to F-actin in a calcium independent manner. Binds to the filaments along their length |
| cytC | DDB0216257 | Cytochrome c; Electron carrier protein. The oxidized form of the cytochrome c heme group can accept an electron from the heme group of the cytochrome c1 subunit of cytochrome reductase. Cytochrome c then transfers this electron to the cytochrome oxidase complex, the final protein carrier in the mitochondrial electron-transport chain (By similarity) |
| erg2 | DDB0267016 | Protein erg2 homolog; May function in lipid transport from the endoplasmic reticulum and be involved in a wide array of cellular functions probably through regulation of the biogenesis of lipid microdomains at the plasma membrane. May regulate calcium efflux at the endoplasmic reticulum (By similarity) |
| gpaG | DDB0185045 | Guanine nucleotide-binding protein alpha-7 subunit; Guanine nucleotide-binding proteins (G proteins) are involved as modulators or transducers in various transmembrane signaling systems |
| hexa1 | DDB0191256 | Beta-hexosaminidase subunit A1; Responsible for the degradation of GM2 gangliosides, and a variety of other molecules containing terminal N-acetyl hexosamines. This enzyme plays a role during the slug stage of development in the maintenance of pseudoplasmodia of normal size; Belongs to the glycosyl hydrolase 20 family |
| mdhB | DDB0230188 | Probable malate dehydrogenase 2, mitochondrial; Catalyzes the reversible oxidation of malate to oxaloacetate; Belongs to the LDH/MDH superfamily. MDH type 2 family |
| nagB2 | DDB0234126 | Glucosamine-6-phosphate isomerase |
| ndkA | DDB0214817 | Nucleoside diphosphate kinase, mitochondrial; Major role in the synthesis of nucleoside triphosphates other than ATP. The ATP gamma phosphate is transferred to the NDP beta phosphate via a ping-pong mechanism, using a phosphorylated active-site intermediate; Belongs to the NDK family |
| nxnA | DDB0232009 | Annexin A7; Calcium/phospholipid-binding protein which promotes membrane fusion and is involved in exocytosis |
| pefA | DDB0191092 | Penta-EF hand domain-containing protein 1; Belongs to the Peflin/Sorcin family |
| proB | DDB0191249 | Profilin-2; Binds to actin and affects the structure of the cytoskeleton. At high concentrations, profilin prevents the polymerization of actin, whereas it enhances it at low concentrations. By binding to PIP2, it inhibits the formation of IP3 and DG |
| psmC1 | DDB0232964 | 26S proteasome regulatory subunit 4 homolog; The 26S proteasome is involved in the ATP-dependent degradation of ubiquitinated proteins. The regulatory (or ATPase) complex confers ATP dependency and substrate specificity to the 26S complex (By similarity). Plays an important role in regulating both growth and multicellular development |
| rab1A | DDB0191476 | Ras-related protein Rab-1A |
| rab5A | DDB0229401 | Ras-related protein Rab-5A; Required for the fusion of plasma membranes and early endosomes |
| rasG | DDB0201663 | Ras-like protein rasG; Ras proteins bind GDP/GTP and possess intrinsic GTPase activity; Belongs to the small GTPase superfamily. Ras family |
| rpl34 | DDB0231151 | 60S ribosomal protein L34; Belongs to the eukaryotic ribosomal protein eL34 family |
| rpl6 | DDB0231338 | 60S ribosomal protein L6; Belongs to the eukaryotic ribosomal protein eL6 family |
| sasA | DDB0214885 | Ras-related protein Rab-8A; Protein transport. Probably involved in vesicular traffic (By similarity) |
| sodC | DDB0232186 | Extracellular superoxide dismutase [Cu-Zn] 3; Protect the extracellular space from toxic effect of reactive oxygen intermediates by converting superoxyde radicals into hydrogen peroxyde and oxygen |
| ssr1 | DDB0266492 | Translocon-associated protein subunit alpha; TRAP proteins are part of a complex whose function is to bind calcium to the ER membrane and thereby regulate the retention of ER resident proteins; Belongs to the TRAP-alpha family |
| vatC | DDB0191419 | V-type proton ATPase subunit C; Subunit of the peripheral V1 complex of vacuolar ATPase. Subunit C is necessary for the assembly of the catalytic sector of the enzyme and is likely to have a specific function in its catalytic activity. V-ATPase is responsible for acidifying a variety of intracellular compartments in eukaryotic cells (By similarity) |

| **Down-regulated processes** |
| --- |
| Actin cytoskeleton |
| Steroid biosynthetic process |
| Oxidation/reduction |
| Lipid metabolic process |
| Other |
| Disconnected node |

**Table S4: α-synuclein down-regulated proteins**

*Proteins with multiple colours involved in more than one process.

| **#node** | **Identifier** | **Annotation** |
| --- | --- | --- |
| DDB0169270 | DDB0234153 | annotation not available |
| DDB0184078 | DDB0184078 | annotation not available |
| DDB0184409 | DDB0235225 | annotation not available |
| DDB0189501 | DDB0233903 | annotation not available |
| DDB0202574 | DDB0202574 | annotation not available |
| DDB0216913 | DDB0305102 | Uncharacterized protein |
| DDB0218146 | DDB0218146 | Uncharacterized protein; Gag |
| DDB0218284 | DDB0218284 | annotation not available |
| DDB0218308 | DDB0218308 | annotation not available |
| DDB0219884 | DDB0233782 | annotation not available |
| DDB0231477 | DDB0231477 | annotation not available |
| DDB0233285 | DDB0233285 | PH domain-containing protein DDB_G0274775 |
| DDB0233381 | DDB0233381 | annotation not available |
| DDB0304688 | DDB0304688 | Poly [ADP-ribose] polymerase; Ankyrin repeat-containing protein |
| H4 | DDB0201644 | Probable glutamine--tRNA ligase; Probable glutaminyl-tRNA synthetase; Belongs to the class-I aminoacyl-tRNA synthetase family |
| abcB2 | DDB0201670 | ABC transporter B family member 2 |
| abcG9 | DDB0214893 | ABC transporter G family member 9 |
| abpA | DDB0191133 | Alpha-actinin A; F-actin cross-linking protein which is thought to anchor actin to a variety of intracellular structures. This is a bundling protein. Increases the actin-stimulated ATPase activity of myosin. Involved in vegetative cell growth, phagocytosis, motility and development, probably through stabilization of the actin network in the cortical cytoskeleton |
| abpF | DDB0230207 | Actin-binding protein F |
| act | DDB0219936 | Actin-related protein 3; Functions as ATP-binding component of the Arp2/3 complex which is involved in regulation of actin polymerization and together with an activating nucleation-promoting factor (NPF) mediates the formation of branched actin networks. Seems to contact the pointed end of the daughter actin filament. The Arp2/3 complex is involved in organizing the actin system in cell motility and chemotaxis, in phagocytosis and macropinocytosis, at late steps of endosome processing, and in mitosis. In concert with a group of other proteins, the Arp2/3 complex plays a general role in [...] |
| adprt1A | DDB0214818 | Poly [ADP-ribose] polymerase |
| aip1 | DDB0214916 | Actin-interacting protein 1; Implicated in both actin filament depolymerization and polymerization. May enhance chemotaxis by promoting cofilin- dependent actin assembly at cell leading edges |
| arcB | DDB0214935 | Actin-related protein 2/3 complex subunit 2; Functions as component of the Arp2/3 complex which is involved in regulation of actin polymerization and together with an activating nucleation-promoting factor (NPF) mediates the formation of branched actin networks. Seems to contact the pointed end of the daughter actin filament. The Arp2/3 complex is involved in organizing the actin system in cell motility and chemotaxis, in phagocytosis and macropinocytosis, at late steps of endosome processing, and in mitosis. In concert with a group of other proteins, the Arp2/3 complex plays a general [...] |
| aspS1 | DDB0231308 | Aspartyl-tRNA synthetase, cytoplasmic 1; Belongs to the class-II aminoacyl-tRNA synthetase family. Type 2 subfamily |
| capC | DDB0219923 | Comitin; May have a role in cell motility. It has high affinity for both G-actin and F-actin. Binds to vesicle membranes via mannose residues and, by way of its interaction with actin, links these membranes to the cytoskeleton |
| carmil | DDB0185176 | Protein CARMIL; Serves as the scaffold for the assembly of a complex that links key players in the nucleation and termination of actin filament assembly with a ubiquitous barbed end-directed motor. This complex is composed of at least capping proteins (acpA and acpB), the Arp2/3 complex, type I myosins (myoB and myoC) and carmil. It has at least a modest ability to activate Arp2/3- dependent actin nucleation. CARMIL localizes along with the Arp2/3 complex, myoB, and myoC in the leading edge of cells and it plays a significant role in the structure and function of these actin- rich cell [...] |
| cnrI | DDB0229864 | Putative countin receptor Cnr9; SET domain-containing protein |
| corA | DDB0191115 | Coronin-A; Required for normal motility. Participates in cytokinesis |
| cpnA | DDB0215368 | Copine-A; Required for cytokinesis, contractile vacuole function and development; Belongs to the copine family |
| crtA | DDB0191384 | Calreticulin; Molecular calcium-binding chaperone promoting folding, oligomeric assembly and quality control in the ER via the calreticulin/calnexin cycle. This lectin may interact transiently with almost all of the monoglucosylated glycoproteins that are synthesized in the ER (By similarity) |
| ddost | DDB0233148 | Dolichyl-diphosphooligosaccharide--protein glycosyltransferase 48 kDa subunit; Essential subunit of the N-oligosaccharyl transferase (OST) complex which catalyzes the transfer of a high mannose oligosaccharide from a lipid-linked oligosaccharide donor to an asparagine residue within an Asn-X-Ser/Thr consensus motif in nascent polypeptide chains |
| erg24 | DDB0232079 | Delta(14)-sterol reductase; Reduces the C14=C15 double bond of 4,4-dimethyl- cholesta-8,14,24-trienol to produce 4,4-dimethyl-cholesta-8,24- dienol; Belongs to the ERG4/ERG24 family |
| fcsA | DDB0191105 | Fatty acyl-CoA synthetase A; Long chain fatty acid acyl-CoA synthetases catalyze the formation of a thiester bond between a free fatty acid and coenzyme A during fatty acid metabolic process. May mediate fatty acid retrieval from the lumen of endosomes into the cytoplasm; Belongs to the ATP-dependent AMP-binding enzyme family |
| fdfT | DDB0231376 | Squalene synthase; Belongs to the phytoene/squalene synthase family |
| gluA | DDB0215373 | Lysosomal beta glucosidase |
| hgsA | DDB0219924 | Hydroxymethylglutaryl-CoA synthase A; Condenses acetyl-CoA with acetoacetyl-CoA to form HMG- CoA, which is the substrate for HMG-CoA reductase |
| myoE | DDB0216200 | Myosin IE heavy chain; Myosin is a protein that binds to actin and has ATPase activity that is activated by actin. May play a role in moving membranes relative to actin |
| napA | DDB0231423 | Nck-associated protein 1 homolog; Involved in regulation of actin and microtubule organization. Involved in cell adhesion |
| omt9 | DDB0266734 | O-methyltransferase 9 |
| pdi2 | DDB0231409 | Protein disulfide-isomerase 2; Participates in the folding of proteins containing disulfide bonds, may be involved in glycosylation, prolyl hydroxylation and triglyceride transfer |
| phesA | DDB0231328 | Phenylalanine--tRNA ligase alpha subunit; Phenylalanyl-tRNA synthetase alpha chain |
| pirA | DDB0216270 | Protein pirA; Involved in regulation of actin and microtubule organization |
| plbA | DDB0185225 | Phospholipase B-like protein A; Phospholipase that removes both fatty-acid chains from phosphatidylcholine and produces the water-soluble glycerophosphorylcholine. In addition to phosphatidylcholine deacylation, it also hydrolyzes phosphatidylinositol and phosphatidylethanolamine |
| pppB | DDB0185058 | Serine/threonine-protein phosphatase PP1; Protein phosphatase activity in vitro; Belongs to the PPP phosphatase family |
| pyd3 | DDB0185221 | Beta-ureidopropionase; Converts N-carbamoyl-beta-aminoisobutyrate and N- carbamoyl-beta-alanine (3-ureidopropanoate) to, respectively, beta-aminoisobutyrate and beta-alanine, ammonia and carbon dioxide |
| racC | DDB0201659 | Rho-related protein racC; Belongs to the small GTPase superfamily. Rho family |
| rps2 | DDB0215391 | 40S ribosomal protein S2; Belongs to the universal ribosomal protein uS5 family |
| sevA | DDB0232954 | Severin; Severin blocks the ends of F-actin and causes the fragmentation and depolymerization of actin filaments in a Ca(2+) dependent manner |
| smt1 | DDB0237965 | Probable cycloartenol-C-24-methyltransferase 1; Catalyzes the methyl transfer from S-adenosyl-methionine to the C-24 of cycloartenol to form 24-methylene cycloartenol |
| snpA | DDB0231538 | Alpha-soluble NSF attachment protein; May be required for vesicular transport between the endoplasmic reticulum and the Golgi apparatus (By similarity). Involved in vesicle fusion with nsfA and probably SNARE proteins; Belongs to the SNAP family |
| svkA | DDB0191176 | Serine/threonine-protein kinase svkA; Involved in regulation of actin cytoskeleton organization during cell motility; F-actin fragmenting and capping protein allowing dynamic rearrangements of the actin cytoskeleton. Also part of a regulatory pathway from the centrosome to the midzone, thus regulating the completion of cell division |
| swp1 | DDB0233147 | Dolichyl-diphosphooligosaccharide--protein glycosyltransferase subunit swp1; Essential subunit of the N-oligosaccharyl transferase (OST) complex which catalyzes the transfer of a high mannose oligosaccharide from a lipid-linked oligosaccharide donor to an asparagine residue within an Asn-X-Ser/Thr consensus motif in nascent polypeptide chains |
| talB | DDB0191526 | Talin-B; Actin-binding protein required for multicellular morphogenesis. Substrate of pkgB and/or pkbA |

| **Up-regulated processes** |
| --- |
| Proteyolysis |
| Positive regulation of RNA polymerase II transcription preinitiation complex |
| Translation |
| tRNA aminoacylation for protein translation |
| Other |
| Disconnected node |

**Table S5: Cotransformant up-regulated proteins**

*Proteins with multiple colours involved in more than one process.

| **#node** | **Identifier** | **Annotation** |
| --- | --- | --- |
| DD7-1 | DDB0238141 | Galactose-binding domain-containing proteinPutative uncharacterized protein DD7-1 |
| DDB0167345 | DDB0238156 | Uncharacterized protein; Short-chain dehydrogenase/reductase family protein; Belongs to the short-chain dehydrogenases/reductases (SDR) family |
| DDB0167437 | DDB0167437 | annotation not available |
| DDB0167945 | DDB0238195 | annotation not available |
| DDB0168140 | DDB0168140 | annotation not available |
| DDB0169073 | DDB0237753 | annotation not available |
| DDB0190011 | DDB0305306 | Obg-like ATPase 1; Hydrolyzes ATP, and can also hydrolyze GTP with lower efficiency. Has lower affinity for GTP |
| DDB0217073 | DDB0234178 | annotation not available |
| DDB0217720 | DDB0217720 | Putative acetyltransferase DDB_G0275913; Belongs to the transferase hexapeptide repeat family |
| DDB0218284 | DDB0218284 | annotation not available |
| DDB0230005 | DDB0230005 | CBS domain-containing protein DDB_G0289609 |
| DDB0230064 | DDB0230064 | annotation not available |
| DDB0252581 | DDB0252581 | Probable GH family 25 lysozyme 5; Belongs to the glycosyl hydrolase 25 family |
| DDB0267102 | DDB0267102 | Probable nucleosome assembly protein; May modulate chromatin structure by regulation of histone octamer formation |
| V4 | DDB0215343 | Vegetative-specific protein V4; Unknown. Its expression during growth is not required for growth but for the proper initiation of development, therefore playing a role in the transition from growth to development |
| abpE-1 | DDB0302489 | Drebrin-like protein; Actin-binding adapter protein. Binds to F-actin but is not involved in actin polymerization, capping or bundling. Does not bind G-actin. Controls pseudopodium number and motility in early stages of chemotactic aggregation |
| ach1 | DDB0233380 | Acetyl-CoA hydrolase; Presumably involved in regulating the intracellular acetyl-CoA pool for fatty acid and cholesterol synthesis and fatty acid oxidation |
| adrm1-1 | DDB0238204 | Proteasomal ubiquitin receptor ADRM1 homolog; Functions as a proteasomal ubiquitin receptor. Recruits the deubiquitinating enzyme uchl5 at the 26S proteasome and promotes its activity (By similarity). Plays a role in the transition from growth to differentiation |
| ap2a1-2 | DDB0302453 | annotation not available |
| argS1 | DDB0231324 | Probable arginine--tRNA ligase, cytoplasmic; Forms part of a macromolecular complex that catalyzes the attachment of specific amino acids to cognate tRNAs during protein synthesis |
| aspS1 | DDB0231308 | Aspartyl-tRNA synthetase, cytoplasmic 1; Belongs to the class-II aminoacyl-tRNA synthetase family. Type 2 subfamily |
| cbp2 | DDB0191196 | Calcium-binding protein 2; Not known; probably binds four calcium ions |
| ccbl | DDB0231138 | Kynurenine--oxoglutarate transaminase; Catalyzes the irreversible transamination of the L- tryptophan metabolite L-kynurenine to form kynurenic acid (KA). Metabolizes the cysteine conjugates of certain halogenated alkenes and alkanes to form reactive metabolites. Catalyzes the beta- elimination of S-conjugates and Se-conjugates of L- (seleno)cysteine, resulting in the cleavage of the C-S or C-Se bond (By similarity); Belongs to the class-I pyridoxal-phosphate-dependent aminotransferase family |
| cct2 | DDB0233992 | T-complex protein 1 subunit beta; Molecular chaperone; assists the folding of proteins upon ATP hydrolysis. Known to play a role, in vitro, in the folding of actin and tubulin (By similarity) |
| cinB | DDB0220110 | Vegetative-specific protein H5; Belongs to the 'GDXG' lipolytic enzyme family |
| dpp3-1 | DDB0266802 | Dipeptidyl peptidase 3 |
| dscA-1 | DDB0266623 | Discoidin-1 subunit A; Galactose- and N-acetylgalactosamine-binding lectin. May play a role in cell-substratum adhesion rather than in cell-cell adhesion. May be necessary for the maintenance of normal elongate morphology during aggregation |
| dscC-1 | DDB0266624 | Discoidin-1 subunit B/C; Galactose- and N-acetylgalactosamine-binding lectin. May play a role in cell-substratum adhesion rather than in cell-cell adhesion. May be necessary for the maintenance of normal elongate morphology during aggregation |
| dscD-1 | DDB0266625 | Discoidin-1 subunit D; Galactose- and N-acetylgalactosamine-binding lectin. May play a role in cell-substratum adhesion rather than in cell-cell adhesion. May be necessary for the maintenance of normal elongate morphology during aggregation |
| dscE | DDB0215382 | Discoidin-2; Galactose-binding lectin. May be necessary for the primary process of spore formation and may be involved in spore coat formation |
| eif3L | DDB0233946 | Eukaryotic translation initiation factor 3 subunit L; Component of the eukaryotic translation initiation factor 3 (eIF-3) complex, which is involved in protein synthesis of a specialized repertoire of mRNAs and, together with other initiation factors, stimulates binding of mRNA and methionyl-tRNAi to the 40S ribosome. The eIF-3 complex specifically targets and initiates translation of a subset of mRNAs involved in cell proliferation |
| eif5 | DDB0234258 | Eukaryotic translation initiation factor 5; Catalyzes the hydrolysis of GTP bound to the 40S ribosomal initiation complex (40S.mRNA.Met-tRNA[F].eIF-2.GTP) with the subsequent joining of a 60S ribosomal subunit resulting in the release of eIF-2 and the guanine nucleotide. The subsequent joining of a 60S ribosomal subunit results in the formation of a functional 80S initiation complex (80S.mRNA.Met-tRNA[F]) (By similarity) |
| eif6 | DDB0234038 | Eukaryotic translation initiation factor 6; Binds to the 60S ribosomal subunit and prevents its association with the 40S ribosomal subunit to form the 80S initiation complex in the cytoplasm. May also be involved in ribosome biogenesis |
| etfa | DDB0267017 | Electron transfer flavoprotein subunit alpha, mitochondrial; The electron transfer flavoprotein serves as a specific electron acceptor for several dehydrogenases, including five acyl- CoA dehydrogenases, glutaryl-CoA and sarcosine dehydrogenase. It transfers the electrons to the main mitochondrial respiratory chain via ETF-ubiquinone oxidoreductase (ETF dehydrogenase) (By similarity) |
| g6pd-1 | DDB0238739 | Glucose-6-phosphate 1-dehydrogenase; Catalyzes the rate-limiting step of the oxidative pentose-phosphate pathway, which represents a route for the dissimilation of carbohydrates besides glycolysis. The main function of this enzyme is to provide reducing power (NADPH) and pentose phosphates for fatty acid and nucleic acid synthesis (By similarity) |
| gluS | DDB0231321 | Probable glutamate--tRNA ligase, cytoplasmic; Catalyzes the attachment of glutamate to tRNA(Glu) in a two-step reaction: glutamate is first activated by ATP to form Glu-AMP and then transferred to the acceptor end of tRNA(Glu); Belongs to the class-I aminoacyl-tRNA synthetase family. Glutamate--tRNA ligase type 2 subfamily |
| gp130 | DDB0214937 | Glycoprotein 130Lipid-anchored plasma membrane glycoprotein 130 |
| gsr | DDB0231410 | Glutathione reductase; Maintains high levels of reduced glutathione in the cytosol |
| hisS | DDB0231332 | Histidine--tRNA ligase, cytoplasmic; Histidyl-tRNA synthetase, cytoplasmic; Belongs to the class-II aminoacyl-tRNA synthetase family |
| hspC | DDB0185048 | 32 kDa heat shock protein |
| hspE-1 | DDB0238264 | Heat shock cognate 70 kDa protein 2; May function in protein folding and assembly, and disassembly of protein complexes |
| leuS | DDB0231253 | Leucine--tRNA ligase, cytoplasmic; Leucyl-tRNA synthetase, cytoplasmic; Belongs to the class-I aminoacyl-tRNA synthetase family |
| ndkB | DDB0238334 | Nucleoside diphosphate kinase, cytosolic; Major role in the synthesis of nucleoside triphosphates other than ATP |
| pgmA | DDB0191348 | Phosphoglucomutase-1; This enzyme participates in both the breakdown and synthesis of glucose; Belongs to the phosphohexose mutase family |
| psmA1 | DDB0214956 | Proteasome subunit alpha type-1; The proteasome is a multicatalytic proteinase complex which is characterized by its ability to cleave peptides with Arg, Phe, Tyr, Leu, and Glu adjacent to the leaving group at neutral or slightly basic pH. The proteasome has an ATP-dependent proteolytic activity; Belongs to the peptidase T1A family |
| psmA4 | DDB0214953 | Proteasome subunit alpha type-4; The proteasome is a multicatalytic proteinase complex which is characterized by its ability to cleave peptides with Arg, Phe, Tyr, Leu, and Glu adjacent to the leaving group at neutral or slightly basic pH. The proteasome has an ATP-dependent proteolytic activity; Belongs to the peptidase T1A family |
| psmC1 | DDB0232964 | 26S proteasome regulatory subunit 4 homolog; The 26S proteasome is involved in the ATP-dependent degradation of ubiquitinated proteins. The regulatory (or ATPase) complex confers ATP dependency and substrate specificity to the 26S complex (By similarity). Plays an important role in regulating both growth and multicellular development |
| psmD1 | DDB0232977 | 26S proteasome non-ATPase regulatory subunit 1; Acts as a regulatory subunit of the 26 proteasome which is involved in the ATP-dependent degradation of ubiquitinated proteins |
| psmD13 | DDB0233004 | 26S proteasome non-ATPase regulatory subunit 13; Acts as a regulatory subunit of the 26S proteasome which is involved in the ATP-dependent degradation of ubiquitinated proteins |
| psmD14 | DDB0191298 | 26S proteasome non-ATPase regulatory subunit 14; Metalloprotease component of the 26S proteasome that specifically cleaves 'Lys-63'-linked polyubiquitin chains. The 26S proteasome is involved in the ATP-dependent degradation of ubiquitinated proteins. The function of the 'Lys-63'-specific deubiquitination of the proteasome is unclear (By similarity) |
| qdpr | DDB0237752 | Dihydropteridine reductase; The product of this enzyme, tetrahydrobiopterin (BH-4), is an essential cofactor for phenylalanine, tyrosine, and tryptophan hydroxylases |
| serS | DDB0231305 | Serine--tRNA ligase, cytoplasmic; Catalyzes the attachment of serine to tRNA(Ser). Is also able to aminoacylate tRNA(Sec) with serine, to form the misacylated tRNA L-seryl-tRNA(Sec), which will be further converted into selenocysteinyl-tRNA(Sec) (By similarity) |
| tbpB | DDB0191435 | 26S proteasome regulatory subunit 6B homolog; The 26S proteasome is involved in the ATP-dependent degradation of ubiquitinated proteins. The regulatory (or ATPase) complex confers ATP dependency and substrate specificity to the 26S complex (By similarity) |
| tkt-1 | DDB0266926 | Transketolase; Catalyzes the transfer of a two-carbon ketol group from a ketose donor to an aldose acceptor, via a covalent intermediate with the cofactor thiamine pyrophosphate |
| trap1 | DDB0185036 | TNF receptor-associated protein 1 homolog, mitochondrial; Chaperone that expresses an ATPase activity |
| valS1 | DDB0231269 | Probable valyl-tRNA synthetase, cytoplasmic; Belongs to the class-I aminoacyl-tRNA synthetase family |
| xpo1 | DDB0234066 | Exportin-1; Mediates the nuclear export of cellular proteins (cargos) bearing a leucine-rich nuclear export signal (NES) |
| zpr1 | DDB0304584 | Zinc finger protein ZPR1 homolog |

| **Down-regulated processes** |
| --- |
| Actin cytoskeleton |
| Steroid biosynthetic process |
| Oxidation/reduction |
| Lipid metabolic process |
| Other |
| Disconnected node |

**Table S6: Cotransformant down-regulated proteins**

*Proteins with multiple colours involved in more than one process.

| **#node** | **Identifier** | **Annotation** | |
| --- | --- | --- | --- |
| DDB0167402 | DDB0238277 | Uncharacterized protein; Belongs to the short-chain dehydrogenases/reductases (SDR) family | |
| DDB0167407 | DDB0167407 | annotation not available | |
| DDB0168319 | DDB0233965 | annotation not available | |
| DDB0168738 | DDB0232205 | annotation not available | |
| DDB0184409 | DDB0235225 | annotation not available | |
| DDB0184511 | DDB0233800 | annotation not available | |
| DDB0186910 | DDB0238597 | annotation not available | |
| DDB0188715 | DDB0234195 | annotation not available | |
| DDB0188980 | DDB0235312 | annotation not available | |
| DDB0189501 | DDB0233903 | annotation not available | |
| DDB0191047 | DDB0302557 | annotation not available | |
| DDB0191714 | DDB0191714 | annotation not available | |
| DDB0191909 | DDB0191909 | Uncharacterized protein | |
| DDB0202574 | DDB0202574 | annotation not available | |
| DDB0204712 | DDB0204712 | annotation not available | |
| DDB0205849 | DDB0235377 | annotation not available | |
| DDB0218053 | DDB0304452 | annotation not available | |
| DDB0218638 | DDB0237522 | annotation not available | |
| DDB0219436 | DDB0233867 | Uncharacterized protein | |
| DDB0231474 | DDB0231474 | Aldehyde dehydrogenase; Belongs to the aldehyde dehydrogenase family | |
| DDB0231475 | DDB0231475 | Aldehyde dehydrogenase; Belongs to the aldehyde dehydrogenase family | |
| DDB0231504 | DDB0231504 | Putative aldehyde dehydrogenase family 7 member A1 homolog; Belongs to the aldehyde dehydrogenase family | |
| DDB0231658 | DDB0231658 | annotation not available | |
| DDB0232204 | DDB0232204 | Aminotransferase class-III; Belongs to the class-III pyridoxal-phosphate-dependent aminotransferase family | |
| DDB0233285 | DDB0233285 | PH domain-containing protein DDB_G0274775 | |
| DDB0233382 | DDB0233382 | Short-chain dehydrogenase/reductase family protein; Belongs to the short-chain dehydrogenases/reductases (SDR) family | |
| DDB0233914 | DDB0233914 | Putative methyltransferase DDB_G0268948 | |
| DDB0234107 | DDB0234107 | annotation not available | |
| DDB0234207 | DDB0234207 | annotation not available | |
| DDB0237843 | DDB0237843 | annotation not available | |
| DDB0266618 | DDB0266618 | SH3 domain-containing protein | |
| DDB0267057 | DDB0267057 | Thimet-like oligopeptidase | |
| aatB | DDB0230093 | Aspartate aminotransferase, cytoplasmic; Plays a key role in amino acid metabolism; Belongs to the class-I pyridoxal-phosphate-dependent aminotransferase family | |
| abcB2 | DDB0201670 | ABC transporter B family member 2 | |
| abpA | DDB0191133 | Alpha-actinin A; F-actin cross-linking protein which is thought to anchor actin to a variety of intracellular structures. This is a bundling protein. Increases the actin-stimulated ATPase activity of myosin. Involved in vegetative cell growth, phagocytosis, motility and development, probably through stabilization of the actin network in the cortical cytoskeleton | |
| abpF | DDB0230207 | Actin-binding protein F | |
| aco1 | DDB0229908 | Probable cytoplasmic aconitate hydratase; Catalyzes the isomerization of citrate to isocitrate via cis-aconitate; Belongs to the aconitase/IPM isomerase family | |
| acpA | DDB0191202 | F-actin-capping protein subunit beta; F-actin-capping proteins bind in a Ca(2+)-independent manner to the fast growing ends of actin filaments (barbed end) thereby blocking the exchange of subunits at these ends. Unlike other capping proteins (such as gelsolin and severin), these proteins do not sever actin filaments | |
| acpB | DDB0191243 | F-actin-capping protein subunit alpha; F-actin-capping proteins bind in a Ca(2+)-independent manner to the fast growing ends of actin filaments (barbed end) thereby blocking the exchange of subunits at these ends. Unlike other capping proteins (such as gelsolin and severin), these proteins do not sever actin filaments | |
| act | DDB0219936 | Actin-related protein 3; Functions as ATP-binding component of the Arp2/3 complex which is involved in regulation of actin polymerization and together with an activating nucleation-promoting factor (NPF) mediates the formation of branched actin networks. Seems to contact the pointed end of the daughter actin filament. The Arp2/3 complex is involved in organizing the actin system in cell motility and chemotaxis, in phagocytosis and macropinocytosis, at late steps of endosome processing, and in mitosis. In concert with a group of other proteins, the Arp2/3 complex plays a general role in [...] | |
| adh5 | DDB0238276 | Alcohol dehydrogenase class-3; Class-III ADH is remarkably ineffective in oxidizing ethanol, but it readily catalyzes the oxidation of long-chain primary alcohols and the oxidation of S-(hydroxymethyl) glutathione | |
| adk | DDB0230174 | Adenosine kinase; ATP dependent phosphorylation of adenosine and other related nucleoside analogs to monophosphate derivatives | |
| adprt1A | DDB0214818 | Poly [ADP-ribose] polymerase | |
| agxt | DDB0237978 | Serine--pyruvate aminotransferase; Dual metabolic roles of gluconeogenesis and glyoxylate detoxification; Belongs to the class-V pyridoxal-phosphate-dependent aminotransferase family | |
| aip1 | DDB0214916 | Actin-interacting protein 1; Implicated in both actin filament depolymerization and polymerization. May enhance chemotaxis by promoting cofilin- dependent actin assembly at cell leading edges | |
| amyA | DDB0214924 | Putative alpha-amylase; AmyA | |
| arcA | DDB0214932 | Actin-related protein 2/3 complex subunit 1; Functions as component of the Arp2/3 complex which is involved in regulation of actin polymerization and together with an activating nucleation-promoting factor (NPF) mediates the formation of branched actin networks. Seems to contact the pointed end of the daughter actin filament. The Arp2/3 complex is involved in organizing the actin system in cell motility and chemotaxis, in phagocytosis and macropinocytosis, at late steps of endosome processing, and in mitosis. In concert with a group of other proteins, the Arp2/3 complex plays a general [...] | |
| arcB | DDB0214935 | Actin-related protein 2/3 complex subunit 2; Functions as component of the Arp2/3 complex which is involved in regulation of actin polymerization and together with an activating nucleation-promoting factor (NPF) mediates the formation of branched actin networks. Seems to contact the pointed end of the daughter actin filament. The Arp2/3 complex is involved in organizing the actin system in cell motility and chemotaxis, in phagocytosis and macropinocytosis, at late steps of endosome processing, and in mitosis. In concert with a group of other proteins, the Arp2/3 complex plays a general [...] | |
| arcC | DDB0201632 | Actin-related protein 2/3 complex subunit 3; Functions as component of the Arp2/3 complex which is involved in regulation of actin polymerization and together with an activating nucleation-promoting factor (NPF) mediates the formation of branched actin networks. Seems to contact the pointed end of the daughter actin filament. The Arp2/3 complex is involved in organizing the actin system in cell motility and chemotaxis, in phagocytosis and macropinocytosis, at late steps of endosome processing, and in mitosis. In concert with a group of other proteins, the Arp2/3 complex plays a general [...] | |
| arcD | DDB0191121 | Actin-related protein 2/3 complex subunit 4; Functions as component of the Arp2/3 complex which is involved in regulation of actin polymerization and together with an activating nucleation-promoting factor (NPF) mediates the formation of branched actin networks. Seems to contact the pointed end of the daughter actin filament. The Arp2/3 complex is involved in organizing the actin system in cell motility and chemotaxis, in phagocytosis and macropinocytosis, at late steps of endosome processing, and in mitosis. In concert with a group of other proteins, the Arp2/3 complex plays a general [...] | |
| arfA | DDB0191101 | ADP-ribosylation factor 1; GTP-binding protein involved in protein trafficking; may modulate vesicle budding and uncoating within the Golgi apparatus | |
| arpA | DDB0220489 | Centractin; Component of a multi-subunit complex, PPK2 (poly P kinase complex 2) involved in microtubule based vesicle motility. It is associated with the centrosome. PPK2 complex can synthesize a poly chain of hundreds of phosphate residues linked by ATP-like bonds | |
| calA | DDB0214955 | Calmodulin; Calmodulin mediates the control of a large number of enzymes, ion channels and other proteins by Ca(2+). Among the enzymes to be stimulated by the calmodulin-Ca(2+) complex are a number of protein kinases and phosphatases | |
| cap | DDB0191139 | Adenylyl cyclase-associated protein; May have a regulatory bifunctional role. Binds G-actin and PIP2. Involved in microfilament reorganization near the plasma membrane in a PIP2-regulated manner | |
| capB | DDB0185023 | cAMP-binding protein 2; Belongs to the CAPAB/TerDEXZ family | |
| capC | DDB0219923 | Comitin; May have a role in cell motility. It has high affinity for both G-actin and F-actin. Binds to vesicle membranes via mannose residues and, by way of its interaction with actin, links these membranes to the cytoskeleton | |
| carmil | DDB0185176 | | Protein CARMIL; Serves as the scaffold for the assembly of a complex that links key players in the nucleation and termination of actin filament assembly with a ubiquitous barbed end-directed motor. This complex is composed of at least capping proteins (acpA and acpB), the Arp2/3 complex, type I myosins (myoB and myoC) and carmil. It has at least a modest ability to activate Arp2/3- dependent actin nucleation. CARMIL localizes along with the Arp2/3 complex, myoB, and myoC in the leading edge of cells and it plays a significant role in the structure and function of these actin- rich cell [...] |
| cmfB | DDB0191095 | Conditioned medium factor receptor 1; Receptor for cmfA, that appears to mediate the G- independent cmfA signal transduction | |
| cnrI | DDB0229864 | Putative countin receptor Cnr9; SET domain-containing protein | |
| coaA | DDB0215369 | Coactosin; Binds to F-actin in a calcium independent manner. Binds to the filaments along their length | |
| cofA | DDB0214987 | Cofilin-1A; Controls reversibly actin polymerization and depolymerization in a pH-sensitive manner. It has the ability to bind G- and F-actin in a 1:1 ratio of cofilin to actin. It is the major component of intranuclear and cytoplasmic actin rods (By similarity) | |
| copb2 | DDB0233798 | Coatomer subunit beta; The coatomer is a cytosolic protein complex that binds to dilysine motifs and reversibly associates with Golgi non- clathrin-coated vesicles, which further mediate biosynthetic protein transport from the ER, via the Golgi up to the trans Golgi network. Coatomer complex is required for budding from Golgi membranes, and is essential for the retrograde Golgi-to-ER transport of dilysine-tagged proteins (By similarity) | |
| corA | DDB0191115 | Coronin-A; Required for normal motility. Participates in cytokinesis | |
| cprD | DDB0214999 | Cysteine proteinase 4 | |
| ctsD | DDB0215012 | Cathepsin D; Protease that may act during cell growth and/or development; Belongs to the peptidase A1 family | |
| cyb5r1 | DDB0266821 | NADH-cytochrome b5 reductase 1; Electron donor reductase for cytochrome b5. The cytochrome b5/NADH cytochrome b5 reductase electron transfer system supports the catalytic activity of several sterol biosynthetic enzymes (By similarity) | |
| cyc1 | DDB0238603 | Cytochrome c1, heme protein, mitochondrial; This is the heme-containing component of the cytochrome b-c1 complex, which accepts electrons from Rieske protein and transfers electrons to cytochrome c in the mitochondrial respiratory chain | |
| cyp508A4 | DDB0232355 | Probable cytochrome P450 508A4 | |
| cysA | DDB0191318 | Cystathionine gamma-lyase; Belongs to the trans-sulfuration enzymes family | |
| ddost | DDB0233148 | Dolichyl-diphosphooligosaccharide--protein glycosyltransferase 48 kDa subunit; Essential subunit of the N-oligosaccharyl transferase (OST) complex which catalyzes the transfer of a high mannose oligosaccharide from a lipid-linked oligosaccharide donor to an asparagine residue within an Asn-X-Ser/Thr consensus motif in nascent polypeptide chains | |
| eapA | DDB0191146 | Alkyldihydroxyacetonephosphate synthase; Catalyzes the exchange of an acyl for a long-chain alkyl group and the formation of the ether bond in the biosynthesis of ether phospholipids | |
| enoA | DDB0231355 | Enolase A | |
| erg2 | DDB0267016 | Protein erg2 homolog; May function in lipid transport from the endoplasmic reticulum and be involved in a wide array of cellular functions probably through regulation of the biogenesis of lipid microdomains at the plasma membrane. May regulate calcium efflux at the endoplasmic reticulum (By similarity) | |
| erg24 | DDB0232079 | Delta(14)-sterol reductase; Reduces the C14=C15 double bond of 4,4-dimethyl- cholesta-8,14,24-trienol to produce 4,4-dimethyl-cholesta-8,24- dienol; Belongs to the ERG4/ERG24 family | |
| fah | DDB0231609 | Fumarylacetoacetase; Belongs to the FAH family | |
| fimA | DDB0214994 | Fimbrin; Binds to actin | |
| fkbp1 | DDB0233549 | FK506-binding protein 1; PPIases accelerate the folding of proteins by catalyzing the cis-trans isomerization of proline imidic peptide bonds in oligopeptides | |
| gabT | DDB0231448 | 4-aminobutyrate aminotransferase | |
| gar1 | DDB0235390 | Probable H/ACA ribonucleoprotein complex subunit 1; Required for ribosome biogenesis. Part of a complex which catalyzes pseudouridylation of rRNA. This involves the isomerization of uridine such that the ribose is subsequently attached to C5, instead of the normal N1. Pseudouridine ("psi") residues may serve to stabilize the conformation of rRNAs (By similarity) | |
| glud2 | DDB0233691 | Glutamate dehydrogenase 2; Belongs to the Glu/Leu/Phe/Val dehydrogenases family | |
| gnd | DDB0215011 | 6-phosphogluconate dehydrogenase, decarboxylating; Catalyzes the oxidative decarboxylation of 6- phosphogluconate to ribulose 5-phosphate and CO(2), with concomitant reduction of NADP to NADPH; Belongs to the 6-phosphogluconate dehydrogenase family | |
| gpaG | DDB0185045 | Guanine nucleotide-binding protein alpha-7 subunit; Guanine nucleotide-binding proteins (G proteins) are involved as modulators or transducers in various transmembrane signaling systems | |
| gpbB | DDB0185122 | Guanine nucleotide-binding protein subunit beta-like protein; Belongs to the WD repeat G protein beta family. Ribosomal protein RACK1 subfamily | |
| gpt | DDB0232139 | Probable alanine aminotransferase, mitochondrial | |
| grxB | DDB0183791 | Glutaredoxin-like protein; Belongs to the glutaredoxin family | |
| gsta1 | DDB0231431 | Putative glutathione S-transferase alpha-1; Conjugation of reduced glutathione to a wide number of exogenous and endogenous hydrophobic electrophiles | |
| hexa1 | DDB0191256 | Beta-hexosaminidase subunit A1; Responsible for the degradation of GM2 gangliosides, and a variety of other molecules containing terminal N-acetyl hexosamines. This enzyme plays a role during the slug stage of development in the maintenance of pseudoplasmodia of normal size; Belongs to the glycosyl hydrolase 20 family | |
| hgsA | DDB0219924 | Hydroxymethylglutaryl-CoA synthase A; Condenses acetyl-CoA with acetoacetyl-CoA to form HMG- CoA, which is the substrate for HMG-CoA reductase | |
| hpd | DDB0231603 | 4-hydroxyphenylpyruvate dioxygenase; Key enzyme in the degradation of tyrosine | |
| hydA | DDB0201650 | Aldehyde dehydrogenase; Belongs to the aldehyde dehydrogenase family | |
| idhC | DDB0231401 | Isocitrate dehydrogenase [NADP] cytoplasmic; Belongs to the isocitrate and isopropylmalate dehydrogenases family | |
| impdh | DDB0230098 | Inosine-5'-monophosphate dehydrogenase; Catalyzes the conversion of inosine 5'-phosphate (IMP) to xanthosine 5'-phosphate (XMP), the first committed and rate- limiting step in the de novo synthesis of guanine nucleotides, and therefore plays an important role in the regulation of cell growth; Belongs to the IMPDH/GMPR family | |
| mai | DDB0231608 | Maleylacetoacetate isomerase | |
| manA | DDB0201569 | Lysosomal alpha-mannosidaseAlpha-mannosidase 60 kDa subunitAlpha-mannosidase 58 kDa subunit | |
| maoA | DDB0231707 | Probable flavin-containing monoamine oxidase A; Belongs to the flavin monoamine oxidase family | |
| metE | DDB0230069 | 5-methyltetrahydropteroyltriglutamate--homocysteine methyltransferase; Catalyzes the transfer of a methyl group from 5- methyltetrahydrofolate to homocysteine resulting in methionine formation; Belongs to the vitamin-B12 independent methionine synthase family | |
| mfeA | DDB0201628 | Peroxisomal multifunctional enzyme A; Enzyme acting on the peroxisomal beta-oxidation pathway for fatty acids. Protects the cells from the increase of the harmful xenobiotic fatty acids incorporated from their diets and optimizes cellular lipid composition for proper development; Belongs to the short-chain dehydrogenases/reductases (SDR) family | |
| mhcA | DDB0191444 | Myosin-2 heavy chain; Myosin is a protein that binds to actin and has ATPase activity that is activated by actin; Belongs to the TRAFAC class myosin-kinesin ATPase superfamily. Myosin family | |
| mppB | DDB0231799 | Mitochondrial-processing peptidase subunit beta; The mitochondrial processing protease (MPP-I) cleaves presequences from mitochondrial protein precursors. Most MPP-I cleavage sites follow an arginine at position -2. mppB is the catalytic subunit of the heterodimeric metallo-endopeptidase. Mitochondrial processing peptidase plays an essential role in mitochondrial biogenesis | |
| mroh1 | DDB0189282 | Maestro heat-like repeat-containing protein family member 1; HEAT repeat-containing protein 7A homolog | |
| myoE | DDB0216200 | Myosin IE heavy chain; Myosin is a protein that binds to actin and has ATPase activity that is activated by actin. May play a role in moving membranes relative to actin | |
| napA | DDB0231423 | Nck-associated protein 1 homolog; Involved in regulation of actin and microtubule organization. Involved in cell adhesion | |
| nxnA | DDB0232009 | Annexin A7; Calcium/phospholipid-binding protein which promotes membrane fusion and is involved in exocytosis | |
| omt9 | DDB0266734 | O-methyltransferase 9 | |
| osbH | DDB0237794 | Oxysterol-binding protein 8; Belongs to the OSBP family | |
| ost1 | DDB0233146 | Dolichyl-diphosphooligosaccharide--protein glycosyltransferase subunit 1; Essential subunit of the N-oligosaccharyl transferase (OST) complex which catalyzes the transfer of a high mannose oligosaccharide from a lipid-linked oligosaccharide donor to an asparagine residue within an Asn-X-Ser/Thr consensus motif in nascent polypeptide chains; Belongs to the OST1 family | |
| pakC | DDB0267078 | Serine/threonine-protein kinase pakC; Has role in the regulation of chemotaxis; Belongs to the protein kinase superfamily. STE Ser/Thr protein kinase family. STE20 subfamily | |
| pckA | DDB0231108 | Phosphoenolpyruvate carboxykinase [ATP]; Belongs to the phosphoenolpyruvate carboxykinase (ATP) family | |
| pdi2 | DDB0231409 | Protein disulfide-isomerase 2; Participates in the folding of proteins containing disulfide bonds, may be involved in glycosylation, prolyl hydroxylation and triglyceride transfer | |
| pefA | DDB0191092 | Penta-EF hand domain-containing protein 1; Belongs to the Peflin/Sorcin family | |
| pepd | DDB0266378 | Xaa-Pro dipeptidase; Splits dipeptides with a prolyl or hydroxyprolyl residue in the C-terminal position; Belongs to the peptidase M24B family. Eukaryotic-type prolidase subfamily | |
| pgl | DDB0231287 | Probable 6-phosphogluconolactonase; Hydrolysis of 6-phosphogluconolactone to 6- phosphogluconate | |
| pirA | DDB0216270 | Protein pirA; Involved in regulation of actin and microtubule organization | |
| pkiA | DDB0216234 | Protein pkiA | |
| pksB | DDB0214951 | PksBShort-chain dehydrogenase/reductase family protein; Belongs to the short-chain dehydrogenases/reductases (SDR) family | |
| plbA | DDB0185225 | Phospholipase B-like protein A; Phospholipase that removes both fatty-acid chains from phosphatidylcholine and produces the water-soluble glycerophosphorylcholine. In addition to phosphatidylcholine deacylation, it also hydrolyzes phosphatidylinositol and phosphatidylethanolamine | |
| ponA | DDB0215380 | Ponticulin; Binds F-actin and nucleates actin assembly. Major high affinity link between the plasma membrane and the cortical actin network | |
| prep | DDB0185041 | Prolyl endopeptidase; Cleaves peptide bonds on the C-terminal side of prolyl residues within peptides that are up to approximately 30 amino acids long; Belongs to the peptidase S9A family | |
| proA | DDB0191178 | Profilin-1; Binds to actin and affects the structure of the cytoskeleton. At high concentrations, profilin prevents the polymerization of actin, whereas it enhances it at low concentrations. By binding to PIP2, it inhibits the formation of IP3 and DG | |
| proB | DDB0191249 | Profilin-2; Binds to actin and affects the structure of the cytoskeleton. At high concentrations, profilin prevents the polymerization of actin, whereas it enhances it at low concentrations. By binding to PIP2, it inhibits the formation of IP3 and DG | |
| prsA | DDB0237882 | Ribose-phosphate pyrophosphokinase A | |
| purD | DDB0230084 | Bifunctional purine biosynthetic protein purDPhosphoribosylamine--glycine ligasePhosphoribosylformylglycinamidine cyclo-ligase; In the N-terminal section; belongs to the GARS family | |
| pyd3 | DDB0185221 | Beta-ureidopropionase; Converts N-carbamoyl-beta-aminoisobutyrate and N- carbamoyl-beta-alanine (3-ureidopropanoate) to, respectively, beta-aminoisobutyrate and beta-alanine, ammonia and carbon dioxide | |
| pyr1-3 | DDB0201646 | Protein PYR1-3; This protein is a "fusion" protein encoding four enzymatic activities of the pyrimidine pathway (GATase, CPSase, ATCase and DHOase) | |
| rac1A | DDB0214822 | Rho-related protein rac1A; Overexpression promotes the formation of filopodia and membrane ruffles; Belongs to the small GTPase superfamily. Rho family | |
| racC | DDB0201659 | Rho-related protein racC; Belongs to the small GTPase superfamily. Rho family | |
| ranA | DDB0215409 | GTP-binding nuclear protein Ran; GTP-binding protein involved in nucleocytoplasmic transport. Required for the import of protein into the nucleus and also for RNA export. Involved in chromatin condensation and control of cell cycle (By similarity) | |
| rasG | DDB0201663 | Ras-like protein rasG; Ras proteins bind GDP/GTP and possess intrinsic GTPase activity; Belongs to the small GTPase superfamily. Ras family | |
| sahA | DDB0191108 | Adenosylhomocysteinase; Adenosylhomocysteine is a competitive inhibitor of S- adenosyl-L-methionine-dependent methyl transferase reactions; therefore adenosylhomocysteinase may play a key role in the control of methylations via regulation of the intracellular concentration of adenosylhomocysteine | |
| sarA | DDB0229965 | GTP-binding protein Sar1A; Component of the coat protein complex II (COPII) which promotes the formation of transport vesicles from the endoplasmic reticulum (ER). The coat has two main functions, the physical deformation of the endoplasmic reticulum membrane into vesicles and the selection of cargo molecules (By similarity) | |
| sasA | DDB0214885 | Ras-related protein Rab-8A; Protein transport. Probably involved in vesicular traffic (By similarity) | |
| sec31 | DDB0235185 | Protein transport protein SEC31; Component of the coat protein complex II (COPII) which promotes the formation of transport vesicles from the endoplasmic reticulum (ER). The coat has two main functions, the physical deformation of the endoplasmic reticulum membrane into vesicles and the selection of cargo molecules (By similarity) | |
| serA | DDB0230052 | D-3-phosphoglycerate dehydrogenase; Catalyzes the reversible oxidation of 3-phospho-D- glycerate to 3-phosphonooxypyruvate, the first step of the phosphorylated L-serine biosynthesis pathway. Also catalyzes the reversible oxidation of 2-hydroxyglutarate to 2-oxoglutarate; Belongs to the D-isomer specific 2-hydroxyacid dehydrogenase family | |
| sevA | DDB0232954 | Severin; Severin blocks the ends of F-actin and causes the fragmentation and depolymerization of actin filaments in a Ca(2+) dependent manner | |
| smt1 | DDB0237965 | Probable cycloartenol-C-24-methyltransferase 1; Catalyzes the methyl transfer from S-adenosyl-methionine to the C-24 of cycloartenol to form 24-methylene cycloartenol | |
| snpA | DDB0231538 | Alpha-soluble NSF attachment protein; May be required for vesicular transport between the endoplasmic reticulum and the Golgi apparatus (By similarity). Involved in vesicle fusion with nsfA and probably SNARE proteins; Belongs to the SNAP family | |
| sodC | DDB0232186 | Extracellular superoxide dismutase [Cu-Zn] 3; Protect the extracellular space from toxic effect of reactive oxygen intermediates by converting superoxyde radicals into hydrogen peroxyde and oxygen | |
| ssr1 | DDB0266492 | Translocon-associated protein subunit alpha; TRAP proteins are part of a complex whose function is to bind calcium to the ER membrane and thereby regulate the retention of ER resident proteins; Belongs to the TRAP-alpha family | |
| swp1 | DDB0233147 | Dolichyl-diphosphooligosaccharide--protein glycosyltransferase subunit swp1; Essential subunit of the N-oligosaccharyl transferase (OST) complex which catalyzes the transfer of a high mannose oligosaccharide from a lipid-linked oligosaccharide donor to an asparagine residue within an Asn-X-Ser/Thr consensus motif in nascent polypeptide chains | |
| thfA | DDB0230118 | Methylenetetrahydrofolate dehydrogenase [NAD(+)]; Catalyzes oxidation of cytoplasmic one-carbon units for purine biosynthesis | |
| trrA | DDB0231235 | Thioredoxin reductase; Belongs to the class-II pyridine nucleotide-disulfide oxidoreductase family | |
| trxE | DDB0237674 | Putative thioredoxin-5; Participates in various redox reactions through the reversible oxidation of its active center dithiol to a disulfide and catalyzes dithiol-disulfide exchange reactions | |
| vasp | DDB0229340 | Protein VASP homolog; Ena/VASP proteins are actin-associated proteins involved in a range of processes dependent on cytoskeleton remodeling and cell polarity such as lamellipodial and filopodial dynamics in migrating cells. Plays a crucial role in filopodia formation, cell-substratum adhesion, and proper chemotaxis. Nucleates and bundles actin filaments. When complexed with fotH in filopodial tips, may support formin-mediated filament elongation by bundling nascent actin filaments; Belongs to the Ena/VASP family | |
